# Innovative optical imaging strategies for monitoring immunotherapy in the tumor microenvironments

**DOI:** 10.1002/cam4.70155

**Published:** 2024-10-10

**Authors:** Um‐e‐Kalsoom, Shiqi Wang, Junle Qu, Liwei Liu

**Affiliations:** ^1^ Key Laboratory of Optoelectronic Devices and Systems of Guangdong Province and Ministry of Education, College of Physics and Optoelectronic Engineering Shenzhen University Shenzhen China

**Keywords:** immune checkpoint, immunosuppression, immunotherapy, optical imaging, tumor microenvironment

## Abstract

**Background:**

The tumor microenvironment (TME) plays a critical role in cancer progression and response to immunotherapy. Immunotherapy targeting the immune system has emerged as a promising treatment modality, but challenges in understanding the TME limit its efficacy. Optical imaging strategies offer noninvasive, real‐time insights into the interactions between immune cells and the TME.

**Objective:**

This review assesses the progress of optical imaging technologies in monitoring immunotherapy within the TME and explores their potential applications in clinical trials and personalized cancer treatment.

**Methods:**

This is a comprehensive literature review based on the advances in optical imaging modalities including fluorescence imaging (FLI), bioluminescence imaging (BLI), and photoacoustic imaging (PAI). These modalities were analyzed for their capacity to provide high‐resolution, real‐time imaging of immune cell dynamics, tumor vasculature, and other critical components of the TME.

**Results:**

Optical imaging techniques have shown significant potential in tracking immune cell infiltration, assessing immune checkpoint inhibitors, and visualizing drug delivery within the TME. Technologies like FLI and BLI are pivotal in tracking immune responses in preclinical models, while PAI provides functional imaging with deeper tissue penetration. The integration of these modalities with immunotherapy holds promise for improving treatment monitoring and outcomes.

**Conclusion:**

Optical imaging is a powerful tool for understanding the complexities of the TME and optimizing immunotherapy. Further advancements in imaging technologies, combined with nanomaterial‐based approaches, could pave the way for enhanced diagnostic accuracy and therapeutic efficacy in cancer treatment.

## Graphical abstract

The tumor microenvironment (TME) is crucial in cancer progression and immunotherapy response. Optical imaging techniques provide a noninvasive, real time method to analyze immune cells in the TME, helping predict and monitor immunotherapy outcomes. This review highlights advancements in optical imaging for TME immunotherapy and its potential in clinical trials and personalized cancer treatment.

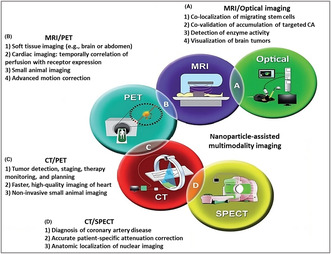

## 1 INTRODUCTION

The immune system plays a vital role in various functions of the body, including disease responses. The cells accumulate to form tumors once the regulation of the everyday life cycle of cells is dysfunctional, and uncontrollable cell proliferation and differentiation occur.^1^, ^2^ Tumors comprise tumor cells, multiple stromal cells, and an extracellular matrix (ECM).^3^ These surrounding cells and noncellular components create the TME, which interacts with the tumor cells and together supports tumor growth and metastasis.^4^, ^5^, ^6^ Compared with tumor cells, micro‐environmental cells have a stable composition, a fixed specialization direction, and are susceptible to environmental factors. These traits have steadily attracted researchers interest.^7^, ^8^, ^9^ Thus, researchers desire to use a specific TME to develop more effective and specific techniques that can achieve satisfactory therapeutic efficacy for cancer progression.^10^, ^11^ For over a century, there has been an ongoing debate about the role of the immune system in the rejection of cancer.^12^ Early clinical trials of immunotherapy were inconsistent; however, recent studies have shown that immunotherapy can be effective in certain patients. This effectiveness mainly arises from the immune system's ability to recognize cancer cells under attack. However, cancer cells can also evade the immune system. Researchers are now developing new immunotherapies that target specific steps in the immune response. The therapies are based on a deeper understanding of how the immune system works and how cancer cells can evade it.^13^, ^14^ Researchers currently consider TME as a basis for defining cancer progression and therapeutic approaches.^15^

A better understanding of the crucial role of TME in modulating the immune response to cancer has led to noteworthy advancements in immune oncology. Diverse immune cells infiltrate the TME, and depending on space and time, they can be intricate in tumor initiation, promotion, progression, and metastasis. The intricacy of TME arises from the regulation of cancer cell proliferation and the impaired development of blood vessels.^16^ Characteristics such as hypoxia, acidic pH, endogenous H_2_O_2_, and modifications in the expression of ECM proteins define the TME, which plays a crucial role in tumor progression and cancer metabolism.^17^ The TME is influenced by both the innate and adaptive immune systems, which play a critical role in body responses to cancer. Current immunophenotyping categorizes the tumor microenvironments (TMEs) into three main types: immune inflamed, immune extended, and immune desert. Immune‐inflamed TMEs are characterized by the presence of immune cells within the tumor, indicating an active immune response and potential for better outcomes with immunotherapy. Immune‐extended TMEs have immune cells presents, but they are trapped in the stroma surrounding the tumor and unable to penetrate it, often due to physical barriers or immunosuppressive factors, which may lead to a less effective immune response. Immune desert TMEs lack significant immune cell infiltration altogether, reflecting an absence of immune activation and typically correlating with poor responses to immunotherapy. Each of these phenotypes has distinct implications for immunotherapy responses. The classification of TMEs is based on the spatial distribution and functional status of immune cells within the tumor and its surrounding stroma. This classification is significant because it helps predict the tumor's responsiveness to immunotherapy and guides therapeutic strategies. For instance, tumor with immune‐inflamed TMEs are generally more responsive to immune checkpoint inhibitors (ICI), while those with immune‐extended or immune desert TMEs may require combination therapies or alternative approaches to overcome the barriers to effective immune cell infiltration and activation. Understanding these classifications enables more personalized and potentially effective treatment plans for cancer patients. Mounting evidence indicates that TME fosters inappropriate metabolic reprogramming of tumor and immune cells. This programming can lead to an immunosuppressive environment that affects nutrient availability, promotes hypoxia, and alters metabolic pathways, affecting T cell function and reducing antitumor immune responses. Addressing these metabolic changes can significantly improve the efficacy of immunotherapies targeting tumors and T cells.^18^, ^19^

Immunotherapy, closely linked to the TME, has proven significant in treating various cancers.^20^ The connection between TME and immunotherapy is essential in determining the effectiveness of immunotherapy interventions. Immunotherapy can also alter TME to improve its efficacy.^21^ Some specific ways the TME can influence the response to immunotherapy are: (1) The physical structure of the TME can affect the response to immunotherapy. The type and number of immune cells in the TME can influence the efficacy of immunotherapy. For example, immunotherapy is likely more effective against cancers with many tumor‐infiltrating lymphocytes (TILs) than against those with fewer TILs.^22^ (2) Tumor cells can activate immune checkpoints to escape the immune system. Immunotherapeutic drugs that block immune checkpoints can trigger anti‐tumor immune responses.^23^ (3) Stromal cells within the TME serve a dual function role in immunotherapy, activating and suppressing antitumor responses.^24^ Thus, immunotherapy can be used to target both supportive and suppressive stromal cells to improve the response. Immunotherapeutic drugs that target stromal cells can improve the response to immunotherapy.^25^ (4) Immunotherapy can cause changes in the tumor vasculature that supply oxygen and nutrients to the tumor. These changes make it more difficult for tumors to grow and spread.^26^ Thus, researchers hope to develop novel immunotherapy strategies that exhibit heightened efficacy and reduced toxicity in patients with cancer.^27^ Immunotherapy can significantly affect the TME by attracting more immune cells to the tumor and enhancing their capacity to eradicate cancer cells. For example, some immunotherapies help to activate these immune cells, increasing their effectiveness in targeting and destroying cancer cells.^28^ By modifying the TME, immunotherapy can create a more hostile environment for cancer cells, impeding their growth and proliferation.^29^

There are three main underlying issues that challenge immunotherapy. First, cancer cells are smart and evasive, usually bypassing the immune system. Second, the tumor suppresses the immune system and poses another challenge for anticancer T cells once the cells are in the tumor. Third, immunotherapy may have adverse effects. Researchers have overlooked the crucial significance of the immune system in tumor therapies for an extended period. This oversight is due to the diverse mechanisms of immunosuppression within the TME, which help tumor progression by inhibiting antitumor responses.^30^, ^31^, ^32^, ^33^ Many checkpoint inhibitors directed at the PD‐1/PD‐L1 pathway have been developed to treat various cancers, such as head and neck cancer, NSCLC, urothelial carcinoma, renal cell carcinoma, and melanoma. Since the approval of ipilimumab for metastatic melanoma treatment in 2011,^34^ the integration of immunotherapies into the management of various cancers has emerged as a viable therapeutic avenue. In some cases, immunotherapies as second‐line treatments for solid tumors offer different ways to combat cancer and can provide enduring long‐term responses.^35^ Despite the encouraging outcomes of immunotherapy, many constraints exist in existing immune biomarkers for prognosticating immune advantages, as in conventional imaging methods for assessing the effectiveness, prognosis, and monitoring of adverse reactions to immunotherapy.^36^ The success of immune checkpoint blockade has underscored the importance of the immune system in cancer treatment. Cancer immunotherapy research aims to enhance T‐cell‐mediated immune responses by mitigating immunosuppression.^37^ Emphasizing strategies that focus on activating intratumoral T‐cell trafficking, coupled with checkpoint blockade, is crucial for optimizing therapeutic efficacy.^29^, ^38^

Optical imaging modalities enhance image contrast and enable real time, high‐resolution TME observations. These modalities encompass various techniques, including fluorescence imaging (FLI), bioluminescence imaging (BLI), intravital imaging (IVI), surface‐enhanced Raman scattering (SERS), and photoacoustic imaging (PA). Optical imaging can study various aspects of the TME, including the following: (1) It can visualize the tumor vasculature, provide information to assess the tumor immune response, and identify potential targets for immunotherapy. (2) It can study stromal cells in the TME, and this information can identify targets for stromal‐based therapies. (3) Optical imaging extends to monitoring drug delivery to the tumor and assessing its distribution within the TME. These data prove instrumental in refining drug delivery strategies and identifying potential targets for combating drug resistance. Imaging biomarkers exhibit a broad spectrum, covering various aspects of TME. Specific imaging of cell types or physiological factors in the microenvironment helps to understand cancer better. It provides important information about how aggressive the cancer is and how it responds to treatment. In vivo imaging plays a pivotal role in tailoring immunotherapies to individual patients, enabling the categorization of individuals poised to derive benefit from immunotherapy, monitoring post‐treatment therapeutic efficacy, and devising alternative strategies for adiaphora.^39^ Optical imaging is undergoing significant advancements as a promising addition to medical imaging, providing biologists and clinicians with innovative approaches for detecting, diagnosing, and studying diseases.^40^ Optical microscopy imaging, which boasts high spatial resolution, has garnered much attention in the past few years in cancer research.^41^, ^42^, ^43^, ^44^ Laser scanning confocal fluorescence microscopy produces high‐resolution 3‐D images. Researchers developed two‐photon fluorescence microscopy with superior spatial resolution and deeper penetration than confocal fluorescence microscopy. Optical coherence tomography has enabled high‐resolution cross‐sectional imaging of tissue scattering.^42^, ^43^ However, their shallow imaging depth limits specific techniques, hindering their nondestructive application in in vivo tumor imaging. Ongoing advancements in optics, light microscopy, and digital imaging have provided a more intricate understanding of the intricate interactions between individual immune system cells and host cells.^45^ Novel imaging methodologies have been employed to observe diverse immune cell types in vivo and assess the response to immunotherapy, encompassing the optimization of the administration route of therapeutic immune cells.^46^, ^47^, ^48^, ^49^, ^50^, ^51^, ^52^ Optical imaging has emerged as a valuable tool for understanding the TME by visualizing cell types and structures to improve our knowledge of tumor growth and develop effective therapies.

A significant hurdle to medical oncology is the creation of effective treatment modalities for advanced cancer. To address distant metastases, therapeutic interventions for advanced primary tumors exhibiting advanced metastases require a concerted approach that combines potent antitumor efficacy for eliminating established tumors with the eviction of a systematic antitumor immune response. The immune system can target entities, adhering specifically to the principle of antigen. Immunotherapy exploits the properties of the immune system to attack and eradicate tumors. The immune system can play an important role in cancer by either promoting tumor growth or fighting it, which can affect survival and prognosis.^53^ Imaging the immune response can provide distinctive insights into cancer immunotherapy mechanisms. Optical imaging has become very significant in cancer imaging^54^, ^55^, ^56^, ^57^, ^58^ and is emerging as one of the most effective imaging modalities for cancer immunotherapy.^59^, ^60^, ^61^, ^62^ A complete scenario of immunological responses was studied via imaging, then used to test immunotherapy and predict future treatment consequences. The primary objective of this review is to provide a comprehensive portrayal of visualizing and comprehending the intricate association between the TME and immunotherapy using various optical imaging techniques, along with their limitations, pitfalls, and promising novel approaches.

## 2 TME

The inherent complexity of tumors continues to pose a challenge to developing effective anticancer therapies.^63^, ^64^ A tumor comprises not only cancerous cells but also complex cancerous and noncancerous cellular structures along with their extracellular milieu, which together form the TME, which comprises an inclusive range of cells, including (1) immune cells. Immune cells contribute to TME. Some can attack and kill tumor cells, such as T cells and natural killer (NK) cells. In contrast, others aid in activating antitumor immune responses, such as macrophages and dendritic cells. T lymphocytes and NK cells kill cancer cells. Macrophages can also destroy cancer cells and polarize them to become pro‐tumor cells, promoting tumor growth and progression. As antigen‐presenting cells, dendritic cells play a pivotal role in triggering antitumor responses. (2) Stromal cells. These cells provide structural support for the tumor and can influence its behavior. These cells include fibroblasts, endothelial cells, and pericytes. Fibroblasts can produce collagen and other ECM components that provide structural support for tumors, making it difficult for drugs to penetrate. Endothelial cells form new vessels, whereas pericytes help stabilize the blood vessels required for tumor growth. (3) Other cells: Other cell types that may be present in the TME include cancer stem cells, circulating tumor cells, adipocytes, neurons, and mesenchymal stem cells (MSCs). MSCs exhibit multipotent characteristics that promote tumor growth and invasion. Adipocytes are fat cells that produce hormones and other signaling molecules that facilitate the growth and progression of tumors. Neurons interact with other cells in the TME to promote tumor growth and progression in different types of brain cancers. Microscopic examination of solid tumors reveals the intricate complexity of cancer, showcasing a highly structured TME.

**FIGURE 1:**
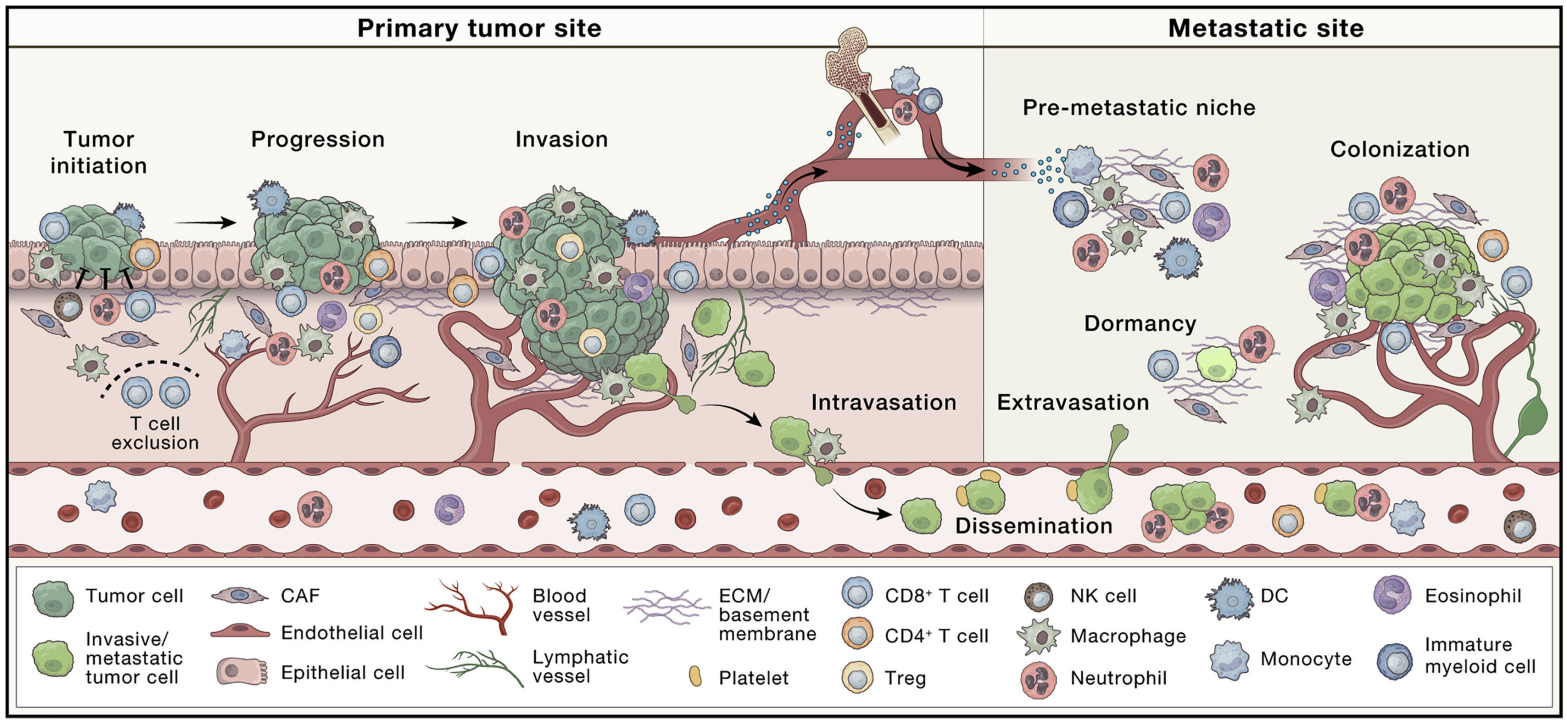
The TME evolves with cancer progression, involving tumor cells (hypoxic or not), lymphatic vessels, blood vessels, adipocytes, mesenchymal stem cells, cancer‐associated fibroblasts (CAFs), pericytes, and immune cells (T and B cells, NK and NKT cells, macrophages, dendritic cells, and neutrophils), among others. Initially, the normal tissue microenvironment restricts cancer growth through suppressive functions of immune cells, fibroblasts, and ECM. However, cancer evade these functions, manipulating the TME to promote tumor growth, invasion, and intravasation. The TME also aids in preparing premetastatic niches, supporting cancer cells survival in circulation, and facilitating metastasis.^71^

By understanding the various types of cells in the TME (Figure 1)^65^, ^66^ their interactions, researchers can develop effective cancer therapies. In tumor growth in metastasis, these cells play a critical role in collectively establishing a complex network. The TME exhibits characteristics such as hypoxia, enhanced vascularization, and reduced pH. Stromal cells and the immune system close to the tumor arrange these phenomena through autocrine and paracrine signaling. Additionally, there is an elevation in interstitial blood pressure and interactions with the immune system.^27^, ^67^, ^68^ The TME governs abnormal tissue functionality and plays a crucial role in the progressive assessment of malignancies.^69^ Autocrine and paracrine communications among TME cells instigate tumor advancement and maturation, resulting in an augmented ECM stiffness, the development of blood and lymph vessels, the potential formation of necrotic regions, and the initiation of metastasis.^68^ This intricate feature activates immune cells within the TME, serving as a barrier that hampers the penetration of drugs into solid tumors.^70^

The TME is a complex network of cells and structures. It surrounds a tumor and plays a key role in its progression. It can promote or suppress tumor growth, invasion, and metastasis. Thus, it could also affect how tumors respond to therapy. Based on the composition of the TME, there are several types of TME, such as:

1. Inflammatory TME: Chronic inflammation characterizes the TME when referred to as an Inflammatory TME. In this type, an imbalance exists between the pro‐inflammatory and anti‐inflammatory signals. Various factors, including tumor cells, tumor‐associated macrophages (TAMs), and cancer‐associated fibroblasts, caused this imbalance. In the TME, signals that causes inflammation can make the tumor grow and spread by increasing cell division, promoting the formation of new blood vessels, and facilitating the spread of cancer cells. Signals that reduce inflammation can help prevent the development of tumors. Immunotherapy was used to boost the antitumor immune response, which can help overcome the immunosuppressive effects of inflammatory TME.
2. Desmoplastic TME: Desmoplastic small round cell tumors (DSRCT) manifest as destructive cancer entities. This describes the presence of compact, small, round cells embedded in a dense fibrous stroma. Treatment of DSRCT usually involves a combination of surgery and therapy.
3. Hypoxic TME: This is a hallmark of solid tumors and occurs when the tumor outgrows its blood supply owing to rapid tumor growth, abnormal blood vessel structure, compression of blood vessels by the tumor, and high metabolic demands of tumor cells. Hypoxia promotes tumor progression, resistance to therapy, and immune invasion.
4. Necrotic TME: This is a TME with many dead tumor cells owing to a lack of oxygen, blood supply, or nutrient deprivation. TME is a challenging environment for cancer immunotherapy because it suppresses the immune system and promotes tumor growth.

**FIGURE 2:**
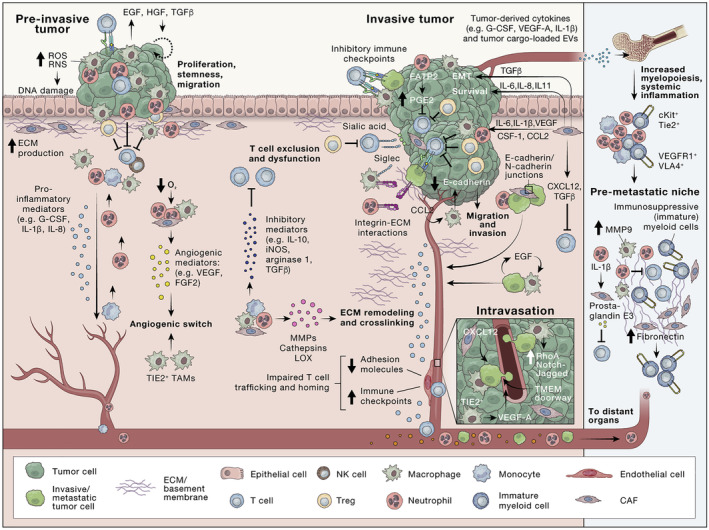
At the earliest stages of tumor initiation, the immune system targets cancer cells for destruction, with fibroblasts and macrophages initially suppressing tumor growth. Overtime, these stromal and immune cells, like TAMs and CAFs, can acquire pro‐tumorigenic functions, promoting angiogenesis and invasion. During intravasation, macrophages assist cancer cells in crossing vessel barriers through TME of metastasis doorways.^71^

The intricate interplay between immune cells, tumors, and diverse environmental stimuli makes up a complex process that dictates the course of either pro‐tumor or anti‐tumor immunity. In the case of acute lymphocytic leukemia, the treatment involves the promising adoptive transfer of T cells endogenously programmed to manifest chimeric antigen receptors (CARs), resulting in a noteworthy up to 90% five‐year survival. However, the application of this treatment is constrained when dealing with solid tumors.^72^ Moreover, the substantial potential of immune checkpoint blockade using antibodies has been demonstrated in shaping tumor immunogenicity, particularly in certain solid tumors such as non‐small cell lung cancer and melanoma.^73^ Response rates to ICIs vary significantly across various tumor types due to the complex nature of the TME.^74^ Advancements in therapeutic strategies hinge upon a comprehensive understanding of TME alterations during tumor development. The mechanisms via which cancer cells cross endothelial layers to enter the circulation are complex, context dependent, and influenced by cancer cell‐intrinsic features, the physical properties of the ECM and type of vasculature, microenvironmental cues, and the extent of hypoxia (Figure 2). The current approach involves targeting many components of the TME. Understanding the pivotal occurrences in the TME that facilitate primary tumor growth and how these events influence the modulation of the environment is crucial. This understanding is paramount in delineating effective therapeutic strategies.^75^

## 3 IMMUNOTHERAPY

The TME plays a crucial role in cancer progression and response to treatment. Some tumors have few inflammatory signals, but others have a significant number of immune cells either around the tumor or inside it.^76^, ^77^ The TME is significant across different cancer types, particularly in terms of immune cell composition. Some tumors exhibit limited inflammatory signals, while others have a substantial presence of immune cells either at the tumor periphery or within its confines. This variability affects how the immune system interacts with tumors, with immune cells acting as both tumor‐suppressing and tumor‐promoting entities, depending on the context. To address these challenges, several immunotherapeutic strategies have been developed. These include: (1) hindering the recruitment of macrophages into tumor tissues; (2) preventing the differentiation of macrophages into the tumor‐promoting phenotype known as TAMs; (3) targeting chronic inflammation or pro‐tumorigenic factors emanating from adaptive immune cells; and (4) stimulating anti‐tumoral responses to combat established tumors.^4^ Immunotherapy aims to enhance and restore the immune system's ability to recognize and eradicate cancer cells through various mechanisms and components designed to stimulate the immune system.^78^

**FIGURE 3:**
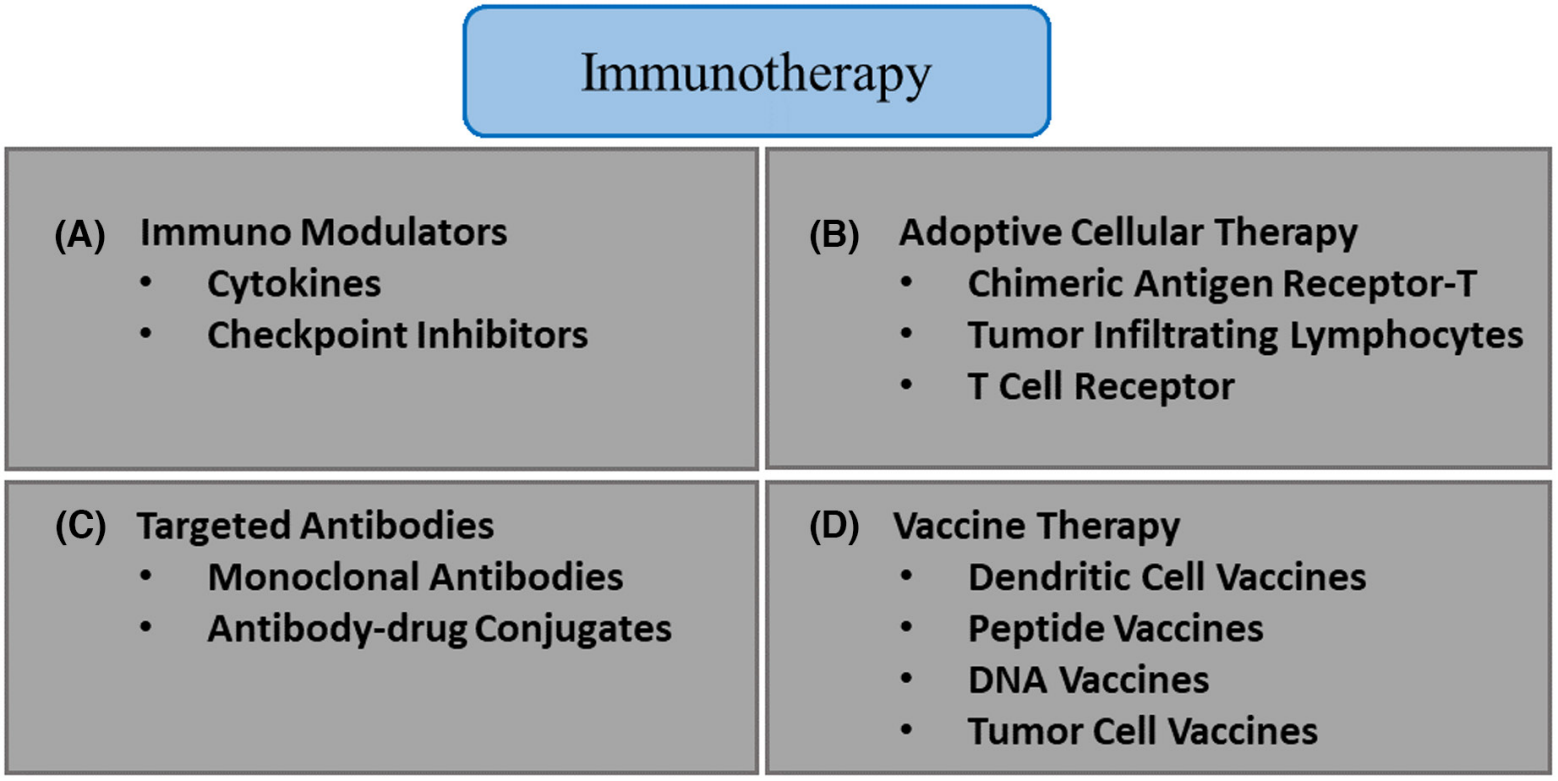
Immunotherapy approaches for cancer treatment.^69^

Researchers have greatly progressed in understanding the complex TME and its role in cancer development. This knowledge has paved the way for innovative immunotherapeutic approaches targeting the TME to augment the immune system's responsiveness to cancer. Various forms of immunotherapy exert distinct mechanisms of action. Specific immunotherapeutic approaches facilitate the immune system by inhibiting or decelerating the proliferation of cancer cells. Alternative strategies empower the immune system to eradicate or regulate the spread of cancer cells to other anatomical regions. Thus, anticancer immunotherapy uses two basic strategies, active and passive, as shown in Figure 3. An active strategy intended to stimulate the patient's immune response involves various methods, such as peptide vaccines, DNA vaccines, oncolytic viruses, dendritic cell‐based vaccines, and immune‐stimulatory cytokines.^79^ In contrast, the passive strategy involves delivering active immune elements to exert direct antitumor effects. This strategy encompasses immunization through antibodies, transfer of in vitro activated cells (e.g., T cells to NK cells), and suppression of immunosuppression. To induce a specific and long‐lasting antitumor immune response, vaccination is an active method to stimulate the immune system. Various types of immunotherapies activate or boost the ability of the immune system to identify and eradicate malignant cells. Recent advances in understanding the TME have led to the development of several immunotherapeutic approaches designed to target and modify the TME to improve the immune response against cancer. Several types of immunotherapies based on these strategies are as follows:

1. Adoptive Cell Therapy: These therapeutic interventions use immune cells to combat cancer via two methodologies. First, isolate, expand, and reintroduce immune cells into patients with cancer for adoptive cell therapy. Second, we reintroduced the genetically modified immune cells into patients with cancer to boost their cancer‐fighting abilities. Different types of adoptive cell therapies are currently available, including TILs, T‐cell receptor (TCR), chimeric antigen receptor (CAR) T‐cell, and NK cell therapies.
2. Cancer Vaccines: These therapies enhance the immune response directed at cancer cells. The similarity between cancer cells and normal cells poses significant challenges for the development of vaccines targeting cancer. Tumor vaccination uses tumor‐associated proteins and peptides as immunogens for dendritic cell vaccination.^78^, ^79^, ^80^ Cancer vaccines include preventive, prophylactic, therapeutic, and personalized neo‐antigen vaccines.
3. Immunomodulators: Molecules that influence pathways regulating immune system activity. These agents modulate immune system responses by either stimulating or suppressing them for cancer treatment. Four groups categorize checkpoint inhibitors, agonists, cytokines, and adjuvants as immunomodulators.
4. Targeted Antibodies: Cancer immunotherapy is designed to disrupt cancer cell activity and prompt the immune system to target and eliminate cancer cells. These antibodies target antigens. The antigens are more abundant on the surface of cancer cells than on normal cells. Some antibodies function as passive immunotherapies, targeting cancer cells without involving immune cells. Active immunotherapies, such as bi‐ and tri‐specific antibodies, need the collaboration of immune cells. Cancer vaccines encompass antibody‐drug conjugates (ADCs), monoclonal antibodies (mAbs), and bi‐specific antibodies.
5. Oncolytic virus therapy: This therapy involves the use of modified viruses in the laboratory to infect and eradicate cancer cells. Certain viruses are designed to infect cancer cells and deliver medicine to them, while minimizing harm to normal cells.

Our understanding of the immune system and its responses to malignant cells has progressively advanced in the last few decades. The field of research on antitumor drugs is developing, and the use of such drugs is increasing, propelled by the rapid advancements in immunotherapy.^81^ Currently, the two main types of immunotherapies are adoptive T cell (ACT) therapy and ICI therapy. ACT involves the removal of the tumor cells by injecting immune effector cells modified by genes.^82^ The immune checkpoint is on the surfaces of T cells or tumor cells that serve as a target to stop the over‐activation of T cells. However, when T cells encounter tumor cells, they are impeded from attacking the tumor, compromising the immune system's ability to identify and eliminate these malignant cells.^83^ A unique advantage of immunotherapy lies in the immune system's capacity to surveil the whole body and eradicate distant metastases as they emerge. Thus, an effective immune response directs its focus with exceptional specificity toward tumors. In recent years, immunotherapy has exhibited notable clinical benefits and holds the potential for achieving long‐term, durable remission.^84^ Despite the lack of a successful endogenous antitumor immune response, immunotherapy has demonstrated potential, commencing with the application of high‐dose IL‐2. IL‐2 represented the sole nonsurgical curative intervention for metastatic melanoma, albeit associated with severe toxicities and a low response rate, till the FDA sanctioned ipilimumab, a monoclonal antibody directed against CTLA‐4, in 2011.^85^, ^86^ During immune response, a subset of the responding T cells undergoes differentiation, giving rise to long‐lived memory cells. These memory cells exhibit two distinct phenotypes: one subset, termed effector memory cells (Tem), takes residence in tissues, while the other, central memory cells (T cm), circulates through secondary lymphoid organs. Both Tem and Tcm can respond to repeated exposures to antigens. In cancer patients, memory CD8^+^ T cells are important, as they are tumor specific. These cells serve a surveillance role against relapse and are postulated to underlie the basis for the observed long‐term, durable cures in certain patients subjected to treatment with anti‐CDLA4 and anti‐PD‐1. These novel immunotherapeutic strategies hold immense potential for revolutionizing tumor treatment. However, currently, few patients experience a sustained remedy, and enhancing this ratio constitutes a pivotal objective within immunotherapy.^87^

## 4 TARGETING TME FOR IMMUNOTHERAPY

**FIGURE 4:**
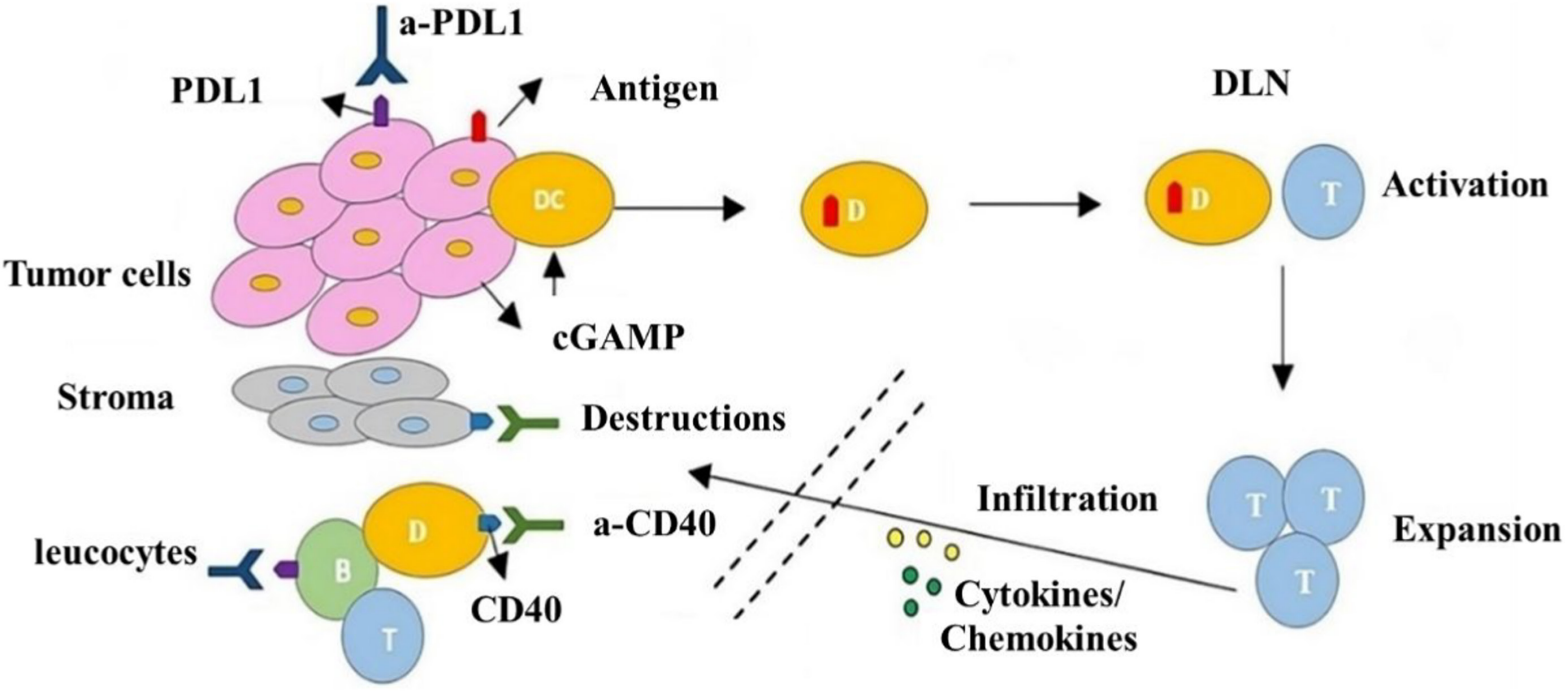
Active tumor control via immunotherapy necessitates initiating immune system activation, infiltration of activated effector cells into the tumor tissue, proliferation of the effector cells, and eradication of tumor cells. Immunotherapy aims to improve those processes, while tumor barriers can dampen them. Through checkpoint molecules, such as PDL1, effector T cells were inhibited, and antiPD1/PDL1 overwhelmed the inhibition via PDL1. Antibodies targeting stimulator checkpoints are employed for eliciting the activation of immune cells; however, for optimized tumor control, some antibodies, for example, anti‐CD40, can also work on stroma cells. The ECM is a barrier, preventing T cells from assessing the TME for tumor damage. The infiltration improves through induction or delivering cytokines or chemokine to the TME. Reproduced with permission from ref.^91^ Copyright 2015, Elsevier.

In immunotherapy, the lack of immune surveillance in people with tumors is a major problem despite advances in treatments like antitumor vaccines and adoptive T‐cell transfer. Interesting evidence from various animal models indicates that when effective antitumor immunity is present, tumors cannot progress, and established tumors undergo regression.^88^ For instance, a study by Galon et al. demonstrated that intratumoral immune cells, particularly CD8+ T cells, strongly correlated with prolonged survival in colorectal cancer patients.^53^ Moreover, recent clinical trials have shown promising results in utilizing checkpoint inhibitors to enhance antitumor immunity. For example, the administration of pembrolizumab, an anti‐PD‐1 antibody, in patients with advanced melanoma resulted in significant tumor regression and improved survival rates. It illustrates that enhancing immune surveillance and overcoming immune suppression within the TME are critical for effective immunotherapy.^89^ Additionally, targeting specific components within the TME has been shown to improve therapeutic outcomes. For instance, targeting regulatory T cells (Treg) has been demonstrated to enhance the efficacy of antitumor vaccines, as evidenced by a study where the depletion of Tregs prior to vaccination led to improved antitumor responses in a mouse melanoma model. Similarly, cytokines such as IL‐2 and IL‐5 have been shown to promote the expansion of effector T cells, enhancing their ability to infiltrate tumors and exert cytotoxic effects.^90^ Furthermore, the role of stromal cells in the TME is being increasingly recognized. A study by Joyce and Pollard highlighted that TAMs promote tumor progression and metastasis through various mechanisms, including the suppression of T cell activity and the promotion of angiogenesis.^90^ Targeting TAMs with agents such as CSF‐1R inhibitors has shown promise in preclinical models, leading to reduced tumor growth and metastasis.^90^ Thus, in contemporary antitumor therapies, the restoration of immune investigation and safeguarding immune cells against tumor‐induced suppression are defined intentions. As our understanding of the molecular mechanism governing tumor escape and induced immune suppression grows, the feasibility of therapeutic interventions to counteract tumor‐induced suppression increases. Future therapeutic strategies adopt a comprehensive approach to reinstating, eliminating tumor escape, antitumor immune responses, and rectifying tumor‐induced immune deviations. This multifaceted strategy is envisioned to empower the host immune system to regulate tumor growth. The efficacy of immunotherapy in achieving tumor control hinges on several key steps, as illustrated in Figure 4:^91^ activation of the immune system, expansion of effector cells, infiltration of activated effector cells into the tumor tissue, and destruction of tumor cells. It is reasonable to suggest that interactions between tumors and surrounding host tissues, especially the leukocyte infiltration, are important in tumor growth. Consequently, interventions that disrupt or modify interactions in favor of the host hold promise for therapeutic benefits. Removing of regulatory T cells before administering antitumor vaccines may enhance their antitumor potential, particularly in patients with advanced malignancies.^92^

Tumors use leukocytes in the TME to support their growth. Scientists are slowly uncovering the specific molecular mechanism behind this. They adeptly evade immune assaults while defending or developing confrontations against immune cells. The nature of inflammatory infiltration differs depending on the local milieu as the tumor advances. Consequently, tumors have developed strategies to neutralize host defenses and evade immune controls across different cancer types, with each tumor's distinct signature manifested in its microenvironment. Consequently, a comprehensive understanding of cellular and molecular interactions within TME is imperative. Transitioning from chronic to acute inflammation at the tumor site holds therapeutic potential. Modern molecular tools enabled the development of innovative and effective anticancer therapies, targeting the tumor and its microenvironment.^92^ Table 1 outlines the elements encompassed by the TME, their impact on the delivery of nanoparticles to solid tumors, and applicable imaging techniques for monitoring these TME factors. The TME reaction to specific treatment modalities is a pivotal consideration in determining the course of treatment continuation.^93^

**TABLE 1:** Summary of TME factors, their impact on drug delivery to tumors, and the methods used to characterize each factor.

TME factors	Effect on tumor drug delivery	Characterization method
Immune cells	Down regulates proapoptotic molecules and upregulates interleukin‐6, protecting tumor cells from chemo‐induced cell death.^77^	Fluorescence, bioluminescence, MRI, PET, ultrasound, CT, and SPECT.
Matrix metalloproteinase	Upregulates anti‐apoptotic molecules protecting cancer cells from chemo‐induced apoptosis.^4^	Optical imaging (Fluorescence, Bioluminescence), PET, SPECT, MRI, PAI, and FMT.
Mesenchymal stromal cells	Can differentiate into various cell types and protect cancer cells from external aggression allowing them to escape apoptosis.^78^	Fluorescence, bioluminescence, MRI, PET, and SPECT.
Tumor vasculature and lymphatics	Modifying the apoptotic signaling pathway and expressing anti‐apoptotic proteins has varied and disorganized blood flow leading to variability in drug distribution.	Fluorescence, bioluminescence, Intravital microscopy, PET, and MRI.
pH	Influences drug uptake based on the acidity of the drug.	Fluorescence, bioluminescence, MRI, PET, and CT.
Tumor‐stroma interactions	The abnormal context of the TME facilitates abnormal cross‐talk allowing tumor cells to disregard rules and adapt to the multicellular environment.^79^	Fluorescence, Intravital microscopy, MRI, and PET.

Abbreviations: CT, computed tomography; FMT, fluorescence molecular tomography; MRI, magnetic resonance imaging; PAI, photoacoustic imaging; PET, positron emission tomography; SPECT, SINGLE‐photon emission computed tomography.

## 5 IMAGING FOR IMMUNOTHERAPY TREATMENT EVALUATION

In the early phases of drug improvement, diverse animal models have been utilized to assess the efficacy of drugs in various disease contexts. For example, many animal models are employed to advance chemotherapeutic and targeted therapies.^94^ In immunotherapy development, conducting experiments is crucial to determining the targeting efficacy and pharmacokinetic and pharmacodynamics properties. Identifying precise biomarkers is important for monitoring immunotherapy and benefiting cancer patients. Significantly, distinctions between imaging biomarkers and conventional tissue‐ or blood‐based biomarkers manifest on multiple fronts. Imaging biomarkers is noninvasive, providing comprehensive information about the entire body, albeit for a single target at a time. These characteristics overcome the limitations associated with sampling and the tissue morbidities linked to conventional tissue/blood biomarkers.^95^

Furthermore, dynamic imaging can provide pharmacokinetic insights. Researching cancer and treatment using imaging technology in preclinical models is an important tool. Within this context, they harbor substantial potential to address the diverse challenges intrinsic to immunotherapy.^96^ To enhance the understanding and application of imaging in immunotherapy, the following questions are critical:

1. Which immune cells are present in the tumor and its microenvironment, and what pivotal role do they play in eliciting a reaction?
2. What functions do the diverse constituents of the TME carry out?
3. What importance underlies the diversity observed within tumors and lesions across various regions?
4. What constitute the biomarkers indicative of an authentic response and genuine progression?
5. In what manner is the correlation elucidated between target expression levels, affinity, and response?
6. Is it feasible to anticipate or identify early resistance?
7. How can in vivo monitoring of the distribution, persistence, and effectiveness of cell‐based immunotherapies be done?
8. Can we predict or detect off‐target effects early? Then, can we predict the resulting toxicities?
9. What strategies can be used for the rational and effective design of combination treatments?

These questions are integral to advancing our understanding and optimizing the use of imaging for immunotherapy evaluation. Metastases can exhibit substantial differences from the primary tumor during tumor assessment, potentially manifesting distinct therapeutic responses compared to the primary lesion. Although anti‐metastatic endpoints are often considered impractical, it is noteworthy that the FDA approved the anti‐metastasis prostate cancer drug apalutamide was used in metastasis free survival as a novel endpoint. This development enhances the outlook for further investigations in the realm of immunotherapy. Consequently, the use of preclinical models employing in vivo traceable cancer cells in the study of metastasis also assumes a pivotal role in advancing immunotherapies. Imaging technology is mandated to provide three‐dimensional information. The feasible parameters amenable to measurement encompass, yet are not defined to, protein expression profiles, gene expression profiles, tissue integrity and pH, vasculature and stromal structure, maps of enzymatic activities, maps of invading immune cell types in response to immunotherapy, and maps of various cell types and subtypes within tumors or TME.

Several imaging modalities have been developed and used in immunotherapy evaluation to track and measure the immune response inside the TME. Every imaging technique has different benefits and drawbacks that affect which applications best suit it. The key characteristics, benefits, and drawbacks of the various imaging modalities used in immunotherapy assessment are compiled in the following table for a thorough comparison: optical imaging, magnetic resonance imaging (MRI), PA, SERS nanomaterial‐based imaging, PA, IVI, and PET as shown in Table 2. This table comprehensively compares different imaging techniques used in immunization assessment, highlighting their principles, advantages, limitations, and specific applications in the context of immunotherapy.

**TABLE 2:** A summary of imaging methods is discussed, including resolution, imaging depth, TME factors, clinical translations, limitations, and applications in immunotherapy that the imaging methods have been used to investigate.

Imaging method	Resolution & depth	TME factors	Clinical translation	Limitations	Applications in immunotherapy
Bioluminescence Imaging	3–5 mm, 1–2 cm	Matrix metalloproteinase, mesenchymal stromal cells, immune cells, tumor vasculature, and pH	Only ideally used as a preclinical technique, human tumors do not express luciferases	Limited to small animal models, and poor spatial resolution. Limited by tissue penetration depth; potential phototoxicity and bleaching	Tracking immune cell migration, gene expression, and tumor growth
Fluorescence Imaging	2–3 mm, <1 cm	Matrix metalloproteinase, mesenchymal stromal cells, immune cells, tumor vasculature, pH, and tumor‐stroma interactions	Translation feasibly lies in fluorescent‐guided surgery, in which clinical trials have been completed	Autofluorescence background, limited tissue penetration. Limited by tissue penetration depth; potential phototoxicity and bleaching	Imaging immune cell distribution and activity, monitoring therapeutic efficacy
Intravital Imaging	100 nm‐1 mm, 100–300 mm	Immune cells, tumor vasculature, and tumor‐stroma interactions	Only feasible for intraoperative guidance	Invasive, requires specialized equipment and expertise, and is limited to small animals	Observing immune cell interactions within the TME, studying cellular dynamics and responses to immunotherapy
Photoacoustic Imaging	5‐300 mm, 0.7–40 mm	ECM proteins, immune cells, tumor vasculature, and pH	The size and cost of laser sources, building a prototype, and conducting clinical trials limit translation	Limited by endogenous contrast; tissue optical absorption and scattering properties	Visualizing oxygenation and vascularization in the TME, monitoring immune cell infiltration, and evaluating therapy response

Abbreviations: ECM, extracellular matrix; TME, tumor microenvironment.

Metastases can exhibit substantial differences from the primary tumor during tumor assessment, manifesting distinct therapeutic responses compared to the primary lesion.^82^, ^83^ Although anti‐metastatic endpoints are often considered impractical, it is noteworthy that the FDA approved the anti‐metastatic prostate cancer drug apalutamide based on metastasis‐free survival as a novel endpoint. This development enhances the outlook for further investigations in the realm of immunotherapy. Thus, preclinical models employing in vivo traceable cancer cells in the study of metastasis also assume a pivotal role in advancing immunotherapies.^96^, ^97^ Imaging technology is mandated to provide three‐dimensional information. The conceivable parameters amenable to measurement encompass, yet are not defined, protein expression profiles, gene expression profiles, tissue integrity and pH, vasculature and stromal structures, maps of enzymatic activities, maps of invading immune cell types in response to immunotherapy, and maps of various cell types and subtypes within the tumor or TME.

## 6 OPTICAL IMAGING TECHNIQUES TO EVALUATE TME FACTORS AFFECTING ANTITUMOR RESPONSE

Electromagnetic radiation, such as light, has built‐in energy that depends on wavelength or frequency. Consequently, light can be use clinically to support diagnosis and treatment.^98^, ^99^, ^100^, ^101^ Many clinical diagnostic imaging modalities, including optical imaging, focus on the interaction between light, tissues, or body fluids.^102^, ^103^, ^104^, ^105^, ^106^ This interaction gives rise to various phenomena, such as scattering, emission, and absorption, providing biochemical and morphological evidence. Clinicians use information from these interactions to evaluate and develop effective treatments for the disease.^107^ Optical Imaging emerges as a sensitive, versatile, and potent instrument for molecular imaging in small animals. Its foundation lies in detecting photons within the visible light spectrum emanating from living tissues, cells, or animals.^108^ Optical imaging stands out as a cost‐effective bio‐imaging method, delivering remarkable in‐plane resolution (50–200 nm)^109^ and high sensitivity with substantial temporal resolution (200–16,000 frames/sec).^110^ Its considerable versatility allows customization according to experimental requirements, and it exhibits the capability to detect low concentrations of target analyte owing to its heightened sensitivity.^110^

**FIGURE 5:**
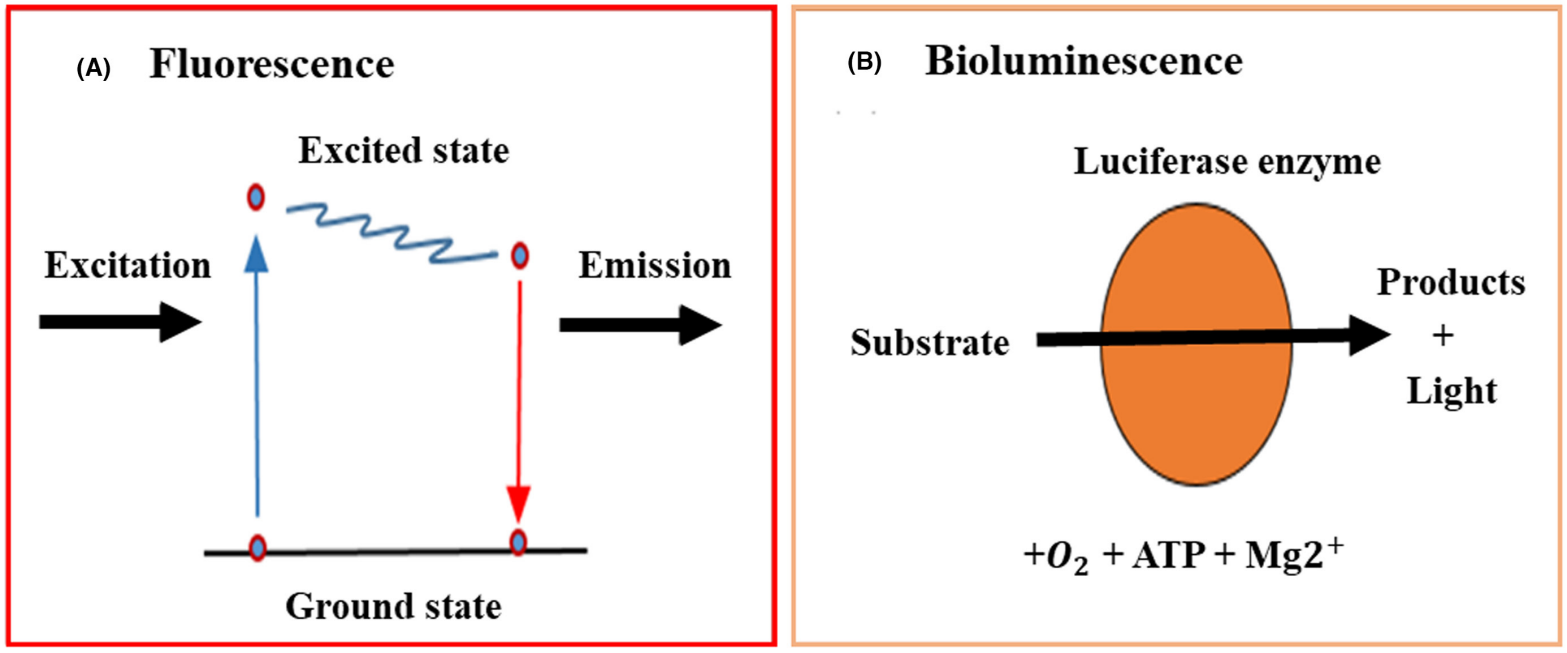
Optical imaging involves the visualization of fluorescent or bioluminescent molecules. (A) Fluorescence happens when a fluorescent molecule is excited and its electrons move to a higher‐energy state. These electrons return to the ground state, releasing energy as emitted light (fluorescence). FLI quantifies the emitted light. (B) Bioluminescence is the result of an enzymatic reaction involving luciferase enzymes. This reaction, powered by cellular ATP and cofactors, produces an oxidized product and visible light. The emitted light was subsequently measured through BLI.

Optical imaging has many uses in biomedical research. Optical imaging is used to study optical signals from cells, tissues, and living organisms. Live in vivo optical imaging proves particularly intriguing as a noninvasive approach for monitoring physiologically induced processes within an individual experimental animal over time. Optical imaging can be categorized into two principal modalities: BLI and FLI, as illustrated in Figure 5. FLI systems offer notable advantages in spatio‐temporal imaging flexibility and the selection of spectral wavelengths. These systems are employed for imaging tumor spheroids with exceptional resolution.^111^ Fluorescent molecules can serve as a source of optical signals, emitting light upon excitation by an external light source (FLI), or luciferase enzymes can generate light as a byproduct of a chemical reaction that transforms an administered substrate (BLI).^112^ The proposed optical imaging (OI) is highly pertinent for monitoring tumor‐specific accumulation and tumor suppression in the course of cancer immunotherapy.^113^, ^114^, ^115^, ^116^, ^117^, ^118^, ^119^, ^120^, ^121^, ^122^, ^123^ Thus, this section reviews the utilization of optical imaging techniques for visualizing the interactions between tumors and immune cells.

### 6.1 BLI

BLI helps study biological processes like tumor growth, metastasis, and in vivo gene expression in small animals.^124^ It offers high resolution and the ability to penetrate deep into tumors, making it valuable for assessing responses to anticancer therapies in preclinical orthotropic animal models.^125^ Unlike other imaging techniques, BLI doesn't rely on external energy sources for excitation, as photons are internally generated^126^, ^127^ through an enzymatic reaction involving the oxidation of a substrate like luciferin or coelenterazine by luciferases.^108^ The normally utilized luciferases, derived from fireflies (*Photinus pyralis*), supply the substrate needed for the light‐producing reaction. However, implementing BLI systems necessitates the transfection or transgenesis of the luciferase gene into cells or animals, along with the administration of substrates before imaging. This approach offers the advantage of regulating luciferase gene expression through a promoter of interest, facilitating protein‐ and cell‐type‐specific examinations using BLI. From the perspective of living cells, the enzymatic reactions of luciferase depend on ATP and oxygen, enabling the use of BLI in cell viability assays.

Nearly four decades ago, NK cells emerged as an encouraging cell type for improving immunotherapy. Solid tumors make it difficult to use NK cell‐based treatment because the immune system in the tumor area can stop the NK cells from working properly. The imperative for optical imaging of NK cells in vivo becomes apparent in the pursuit of novel therapies, enabling real time evaluation of therapeutic responses and identification of off‐target effects.^128^ Using BLI, researchers examined the bio‐distribution of NK‐92MI cells, a derivative of the NK‐92 cell line. BLI was conducted on NK‐92MI cells localized within a pulmonary metastatic model derived from the CAL‐62 cell line. Luciferase‐expressing NK092MI cells were administered to mice through the tail vein, and imaging was executed 48 hour's post‐injection. The results showed that NK‐92MI cells migrated to metastatic sites and reached peak intensity at 24 hours.^129^ BLI reporter systems are suited for biomedical research owing to their low background signal, high signal‐to‐noise ratio, noninvasive nature, brief acquisition time, and the ability to monitor multiple animals together.^108^ BLI has been instrumental in monitoring tumor cell growth and regression,^130^, ^131^ visualizing kinetics of tumor cell clearance through therapeutics, and tracking gene expression.^132^, ^133^ BLI stands as the predominant preclinical methodology for investigating the influence of immunotherapeutic on visible bioluminescent tumors in vivo. In the realm of preclinical immunotherapy advancements, BLI provides distinctive evidence; for instance, the dual‐luciferase reporter method facilitates the quantification of in vivo T cell activation in engineered transgenic mice.^134^, ^135^

### 6.2 FLI

In FLI, external light sources excite fluorophore with specific wavelengths, allowing visualization and quantification of diverse cell types in the TME, including tumors, immune cells, and stroma cells.^108^ FLI allows for the monitoring of administered fluorescent molecules, which need external light stimulation for excitation. Upon absorbing the incoming photon's energy, they emit lower‐power photons, that is, higher‐wavelength photons, forming the FLI signal. In whole‐body imaging, where optical signals may originate from deep tissues, photons must traverse emission and excitation processes. NIR dyes, with wavelengths slightly higher than the human visible spectrum, are used for in vivo FLI, as they are less likely to get absorbed by tissues. Fluorescent dyes for optical imaging include those that emit strong signals when exposed to light, such as pH probes, bio‐reductive and activity‐based probes, and oxygen‐sensitive probes.^136^ In vivo optical imaging applications have utilized diverse NIR imaging fluorophore, with fluorescent probes and labels serving as the predominant tools. Notable examples include Kodak X‐SIGHT dyes and Conjugates, Alexa Fluor 680 and 750 Dyes, IRDye 680, and 800CW Fluors, and DyLight 750 and 800 Fluors, Pz 247. Numerous studies have substantiated the use of infrared dye‐labeled probes in optical imaging. For instance, in targeting diverse cancers, NIR‐labeled RGD targeting αvβ3‐integrin has been extensively investigated.^137^ Additionally, in the context of tumor progression imaging, a NIR fluorophore has been associated with EGF.^138^ Comparisons among a NIR fluorophore and Cy5.5 have demonstrated that extended wavelength dyes yield supplementary active targeting agents for optical imaging.^139^ Immunotherapy stands out as an impressive and effective approach for exploiting the inherent capabilities of the patient's immune system to eradicate cancer cells. The observation of distinct immune cell activities in vivo yields substantial evidence, contributing to the enhancement of immunotherapy therapeutic outcomes.^30^, ^142^, ^143^ In ref,^33^ the noninvasive two‐color fluorescence molecular imaging of a crucial immune cell known as myeloid‐derived suppressor cells (MDSCs) was successfully achieved in vivo. This was accomplished by utilizing NIR‐II (1000–1700 nm) nanoprobes. MDSCs were recognized in vivo via two‐color molecular imaging because of the QD‐based nanoprobes molecular targeting and nonoverlapping emission abilities of QD‐based nanoprobes. MDSC distribution was revealed in different organs and tissues, aided by the reduced auto‐fluorescence and suppressed photon scattering for the NIR‐II window, which was unachievable noninvasively via other methods. Noninvasive high‐resolution imaging of the tumor site was attained owing to the high signal‐to‐background ratio and clarity in the NIR‐II‐b area, which confirmed the presence of MDSC clusters. Anti‐tumor immune response was assessed by observing the quantity of MDSCs in the tumor immune microenvironment following drug treatment. The imaging platform, centered on nanoprobe technology, provides visual data to examine dynamic alterations in immune cell populations within the tumor immune microenvironment. This capability aids in informing clinical medicine decisions and evaluating therapeutic efficacy.^140^

**FIGURE 6:**
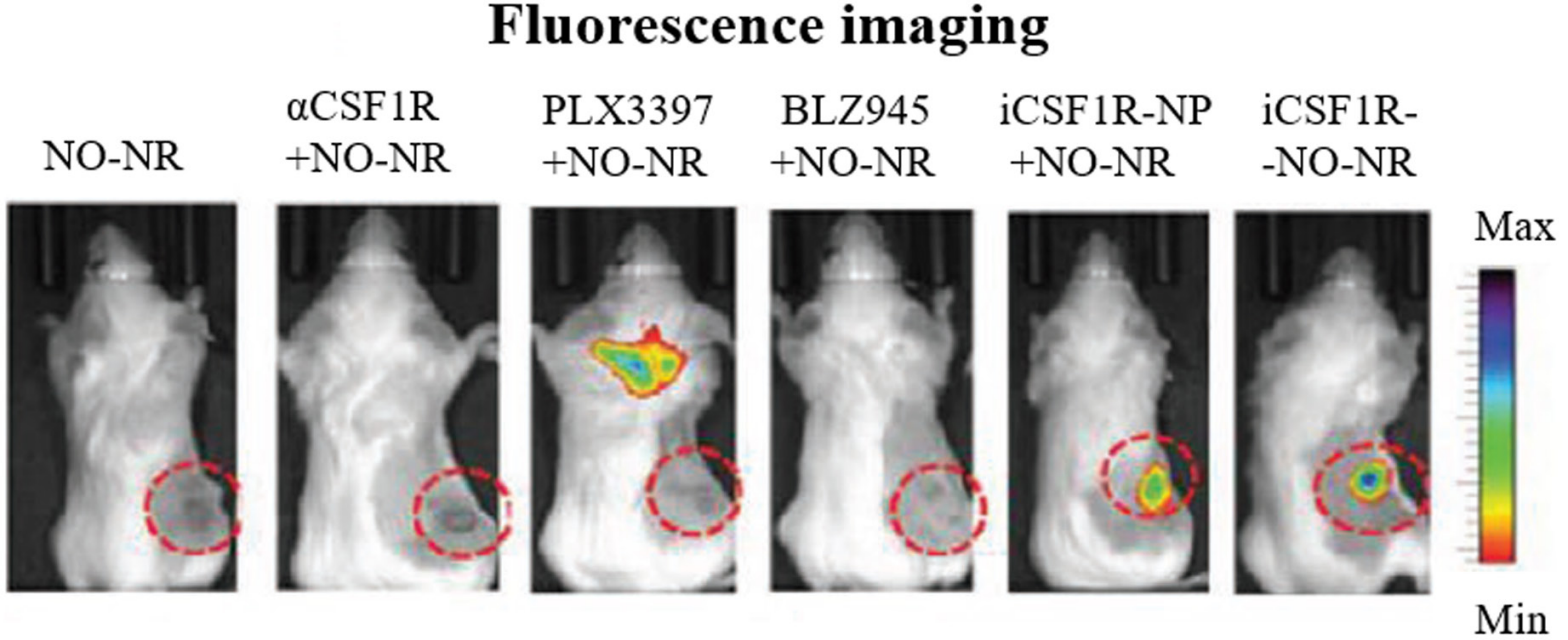
Fluorescence imaging was employed to observe the dynamic activity of nitric oxide in macrophages undergoing treatment with various immunotherapy drugs designed for macrophage modulation in real time. Adaptation with permission from ref.^141^ Copyright 2020, Wiley‐VCH.

A study by Brouillard et al. introduced a nitric oxide nanoreporter (NO‐NR) for real time monitoring of immunotherapy drug effects on macrophages in vitro and in vivo, as shown in Figure 6.^141^ Tests on the NO‐NR system revealed insights into the relationship between macrophage reprogramming imaging and drug‐free treatment, suggesting NO imaging as a favorable technique for real time prognosis of macrophage‐based immunotherapy. Another study demonstrated concurrent measurement of TAM content, protease activity, and integrin expression through imaging,^142^ advancing the identification of new predictors for a comprehensive understanding of tumor responses to therapy.^143^

A limited number of investigations have documented the use of fluorescent imaging to observe NK cells or NK cell lines. Lim et al. used an anti‐CD56 antibody coated by QD705. QD705 is a quantum dot that emits in the NIR region and they use it to label NK‐92MI cells. Quantum dots, due to their reduced cellular internalization, prove advantageous for NK cell imaging.^128^ Administration of NK‐92MI cells transpired on two distinct days, with imaging conducted the day following the second intertumoral injection. The presence of NK cells in tumors has been confirmed, and mice receiving NK cells exhibited observable tumor regression. QD705 was found to have minimal toxicity to NK cells, as shown in a study by analyzing cell viability using fluorescence‐activated cell sorting.^144^ In 2016, Samit et al. introduced an imaging probe, NIR‐PD‐L1‐mAb, designed for detecting PD‐L1 expression. Elevated fluorescence signal intensities have been observed in MDA‐MB‐231 tumors matched to SUM149 tumors (27% vs. 0.1% PD‐L1‐positive cells). This investigation demonstrated the capability of NIR‐PD‐L1‐mAb to identify PD‐L1 expression through FLI.^145^ In 2017, Du et al. utilized the imaging probe PD‐1‐IRDye800CW, revealing its precise accumulation in the tumor region, which was 1.7‐fold higher than that of IgG. These findings further corroborated the viability of PD‐1‐IRDye800CW for detecting PD‐L1 expression via fluorescence optical imaging.^36^, ^146^

FLI allows precise monitoring of gene expression, tumor growth and metastasis, angiogenesis, and bacterial infection in a quantitative manner.^147^ Fluorophore utilization in imaging is influenced by factors such as emission wavelength, excitation wavelength, and quantum yield, each of considerable importance.^112^ FLI has proven valuable in observing tumor burden and metastasis seeding,^148^, ^149^, ^150^ delineating tumor architecture and cellular heterogeneity,^151^, ^152^, ^153^ characterizing the TME,^154^ and evaluating therapeutic responses in both animal models of cancer and patient‐derived xenografts.^148^, ^155^

### 6.3 IVI

**FIGURE 7:**
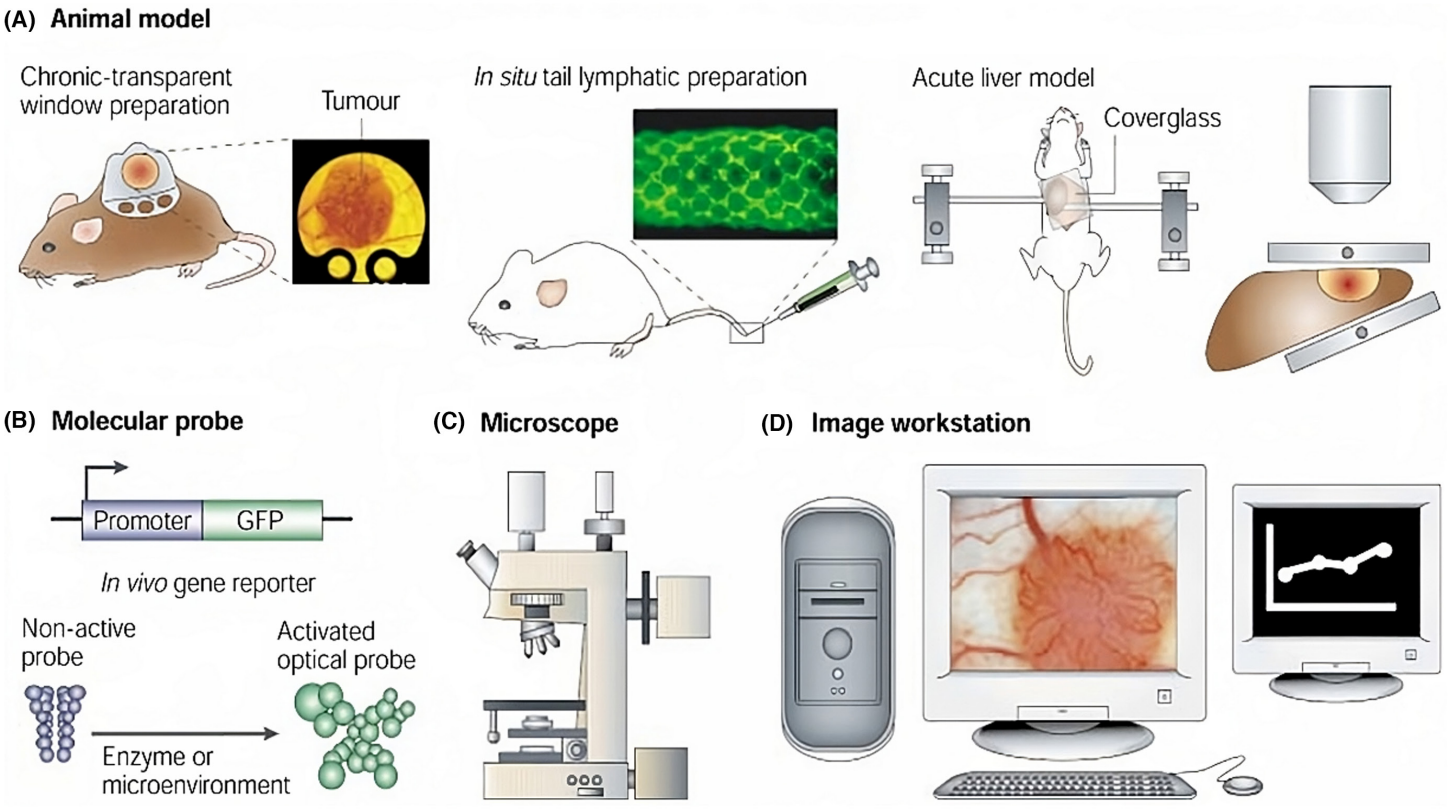
Intravital microscopy involves a sequence of four steps. (A) Selecting an appropriate model: (1) Using a transparent window on the skin where the tumor grows, allowing observation through a coverslip; (2) injecting an optical tracer into the tail interstitial space for an in‐situ preparation; (3) using a surgical preparation to expose a tumor within the liver. (B) Entails the use of a molecular probe. Two illustrative examples were presented: The first involves a green fluorescent protein (GFP) articulated in animals or cells that are genetically engineered and regulated through a promoter of interest; the second features an optical probe actuated through a specific enzyme. (C) A microscope equipped with an excitation source and detection system is required. This system captures images that are transmitted to a computer acquisition and analysis workstation. (D) Involves an image workstation where sophisticated image processing and analyzing algorithms are employed to extract quantitative data from the acquired images.^157^

Intravital microscopy (IVM) is a potential optical imaging method used to observe tissues at subcellular resolution (1–10 μ) in live animals. Achieving such a resolution is unattainable through nonoptical approaches.^156^ IVI facilitates continuous observation of the dynamic immune system and monitoring of cellular recruitment within live animals. IVI has contributed an important part in revealing insights into tumor‐associated vasculature and the involvement of effector immune cells within the TME. There are two options for assessing therapeutic responses in tumors, as depicted in Figure 7, first involves invasive techniques that cause tissue excision, while the second entails noninvasive techniques characterized by lower spatial resolution (mm).^157^ Constraints limit the ability to capture temporal dynamics and images at the subcellular level. IVI offers an understanding of diverse molecular and cellular processes with exceptional spatial and temporal resolutions at subcellular levels.^158^ This has enabled the development of many cell types, such as monocytes, T cells, macrophages, and neutrophils, in the TME. Intravital microscopic approaches play an important role in broadening insights into antitumor immune response within TME, particularly in the context of monitoring tumor regression.^159^ Studying tumor cell reactions to immunotherapy in an intact microenvironment in mice through IVM provides an exceptional opportunity to gain better insights into the complex interactions that lead to therapy resistance.^156^

**FIGURE 8:**
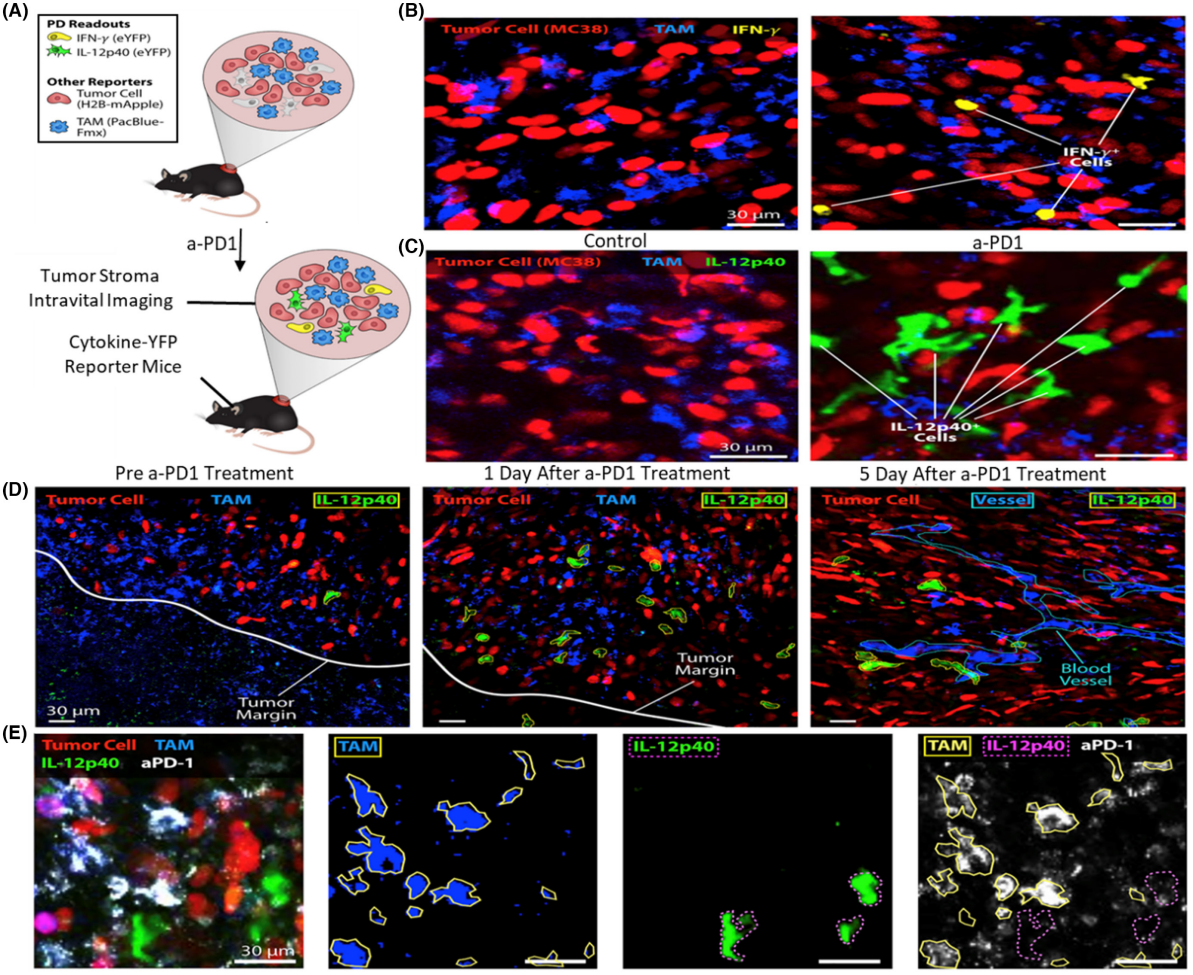
Treatment with aPD‐1induces the secretion of IFN‐γ and IL‐12 inside the TME (A) Intravital imaging employed to monitor pharmacodynamics in MC38‐H2B‐mApple tumor‐bearing mice, focusing on the dynamics of myeloid cell and lymphoid post‐aPD‐1 treatment. (B) Depicts tumor imaging in reporter mice (IFN‐γ‐eYFP) with and without aPD‐1 treatment. Tumor cells (red), TAM (pacific blue), and IFN‐γ‐eYFP (yellow). (C) and (B) show images from IL‐12p40‐eYFP reporter mice. The images highlight IL‐12p40‐eYFP (Green), tumor cells (red), and TAM (blue). (D) Imaging was conducted before and after the aPD‐1 treatment, with visualization of tumor vessels using Pac Blue‐labeled dextran. Different elements, such as tumor margin, tumor cells, IL‐12+ cells, TAM, and blood vessels, are denoted by white, red, green, blue, and cyan colors. (E) It provides intravital images of MC38 TME in an IL12 reporter mouse 5 days after AF647‐aPD‐1 therapy (in vivo). The elements are aPD‐1 (white), tumor cells (red), TAM (blue), and IL‐12p40 (green). Reproduced with permission from ref.^162^ Copyright 2018, Elsevier.

Cuccarese et al. investigated uneven dispersion of macrophages within tumors, revealing significant variability across different tumor sizes. This heterogeneity is linked to the ineffectiveness of drug delivery in nontherapeutic contexts. The researchers witnessed that observation of nanotherapeutics into the tumor was contingent upon macrophage density, and the reduction of macrophages resulted in diminished delivery and efficacy of nano therapy.^160^ The IVM method has been employed for scrutinizing the interaction between dendritic cells and CD8+ tumor‐infiltrating T cells within the immunosuppressive TME.^161^ This real time imaging method has enabled the monitoring of pivotal roles played by immune response participants in TME, specifically focusing on IFN‐ɣ and IL‐12. To observe these key mechanisms, the expression of IL (IL‐12p40‐IRES‐YEP) and IFN‐ɣ (IFN‐ɣ‐IRES‐YEP) was evaluated using reporters. Pacific blue dextran NP and H2B‐mApple were utilized to label macrophages and tumor cells (MC38), facilitating the visualization of the TME as illustrated in Figure 8A.

In the TME, a notable enhancement in the emergence of IL‐12p40‐eYFP^+^ and IFN‐ɣ‐eYFP^+^ cells was noticed within 1 day of aPD‐1 treatment, suggesting a heightened buildup of DCs in tumors, as illustrated in Figure 8B–D. The activation mechanism of DCs involved aggregation of aPD‐1 in TAMs rather than in IL‐12 + DCs, as depicted in Figure 8E. DCs contributed to the efficacy of aPD‐1 treatment and the crosstalk between DCs and T cells was assisted through IL‐12 and IFN‐ɣ.^162^ Imaging studies have exposed that tumor‐infiltrating DCs generated from IL‐12 have a significant influence on checkpoint blockade, contributing to the elicitation of an anti‐tumor response. NK cell recruitment plays an important role in enhancing patient responsiveness to checkpoint blockade, thereby improving total survival. The correlation between NK cell frequency and inter‐tumoral stimulatory dendritic cells is associated with an augmented response to aPD‐1 immunotherapy.^163^ Imaging data explain the tumor escape mechanism, demonstrating that checkpoint blockade can enhance CTL activity within lymph nodes.^164^ Real time imaging was employed to investigate the subcellular activity of aPD‐1. Kohler et al. used a method to track immune and tumor cells simultaneously by using dyes, Pacific Blue‐dextran NPs, and fluorescent proteins (GFP and YFP). The inhibitory effect of aPD‐1 on tumor growth was evident. Examination of imaging data disclosed that aPD‐1 initially entered tumor vessels and diffused into the tumor interstitium. In contrast, TAMs internalized AF647‐ aPD‐1, with no observed interactions at these points. T cells transferred the AF637 signal, and this happened over short (15 min) and long‐term (24 h) durations. In vivo experiments have demonstrated that preadministration blockade of the FCɣ receptor prolongs aPD‐1‐T cell interactions.^165^

An additional investigation delved into the involvement of TAMs in immune activation mediated by T cells, revealing that the interactions among TAMs and CD8 + T cells persisted for an extended duration within the tumor stroma. Following TAM depletion or blockade of TAM:CD8 + T cell interactions, researchers observed an increase in interactions between the tumor and T cells, along with an increased presence of tumor‐infiltrating CD8 + T cells. The efficacy of an aPD‐1 cancer immunotherapy demonstrated enhancement upon macrophage depletion, resulting in a diminished macrophage‐mediated exclusion of T cells.^166^ Ensuring a cytotoxic CD8 + T cell‐mediated antitumor response relies on stable interactions between lymphoma cells and T cells.^167^ CD8 + T cells accumulate in tumor stroma. Thus, the presence of ECM^168^ and tumor hypoxia^169^ exerts a negative influence on T cell migration, hindering antitumor response. IVM, employed to visualize TAM migration, has uncovered protease‐dependent migration modes within mouse breast cancer and fibrosarcoma. This observation suggests that TAMs can be targeted by regulating their migration.^170^ Following combination immunotherapy, including ACT and cyclophosphamide, IVI of crucial cellular processes substantiated the activation of innate antitumor responses, leading to the elimination of the immunosuppressive environment. Additionally, infiltration of endogenous DCs and CTLs into the tumor was observed.^171^

Intravital multiphoton microscopy enables the study of cells and cellular processes inside the body using a surgically implanted imaging window.^172^ This impactful technique revolves around excitation and emission wavelengths to investigate specific fluorescent probes, absorbing two low‐energy photons, thereby enhancing tissue penetration, minimizing damage, and producing a clearer image.^173^ The advantage lies in the ability to visualize the dynamics of the TME in three dimensions at the single‐cell level.^174^ Research using this method has unveiled intricate cellular dynamics of CARs T cells and their effectiveness in combating tumors.^167^, ^175^ However, a limitation of this technique is the potential connection among correlated fluorophores, particularly after employing specific laser light source and microscope filter sets to label and image multiple components of TME. To address particular constraints, fluorescence‐lifetime imaging microscopy (FLIM) is employed, where fluorophores having diverse fluorescence lifetimes allow the exploration of TME dynamics.^93^, ^176^ The studies discussed in this review article illustrate the success of optical imaging in the realm of cancer immunotherapy, underscoring the crucial role of imaging in visualizing and monitoring therapeutic efficacy while eliciting antitumor immune responses.

### 6.4 PA of Immunotherapy

PA is an emerging hybrid imaging method that combines optical and ultrasonic imaging to offer high‐resolution deep‐tissue imaging. The photoacoustic (PA) effect is derived from the acoustic wave, which provides biomolecules that are excited optically, and allow in‐depth imaging of biological tissues with minimum signal loss in vivo.^177^ It is valuable in cancer immunotherapy as it can visualize molecular and cellular changes in the TME in real time. Ultrasonic transducers, resulting in detailed images, then detect these waves. This technique has several advantages: it provides higher spatial resolution, deeper tissue penetration than traditional optical imaging methods; it enables the visualization of specific biomarkers, functional parameters, such as oxygen saturation and hemoglobin concentration, which are essential for monitoring the TME. A dextran‐based pH‐sensitive NIR nanoprobes was designed and used as a contrast agent for PA for detecting tumors in vivo. The nanoprobes with pH‐sensitive dual NIR resonance absorption showed significant pH‐dependent PA signals. Nanoprobes have been shown to concentrate in breast tumors in a mouse model, as examined by dual‐wavelength PA. Normal tissues have not been affected.^178^ A PA probe that induces caspase‐3 has been created for detecting tumor apoptosis in vivo. In this study, a caspase‐3‐recognized PA probe was cleaved to facilitate macrocyclization, which is followed by self‐assembly, resulting in tumor‐specific PA signal amplification. The prolonged retention of self‐assembled probes in tumors allowed for observation of caspse‐3 activity and distribution throughout the tumor following doxorubicin (DOX) therapy.^179^ Wu et al. used a similar method using enzyme‐activatable probes to create a PA probe for visualizing alkaline phosphatase (AP) activity in vivo. In the presence of AP, the PA probe dephosphorylates, generating a hydrophilic‐to‐lipophilic transition that enhances the signal. In vivo PAI in HeLa malignancies revealed that the probe attained a peak contrast ratio of 2.3 times (experimental and control) after 4 hours after delivery.^180^

Monitoring MMP synthesis in the TME can serve as a biomarker for metastasis, as demonstrated by preclinical studies using PAI.^181^, ^182^ Exogenous contrast agents can mark and monitor immune cells in vivo, as demonstrated using fluorescently tagged T cells.^183^, ^184^ Various contrast agents, including as organic semiconducting polymer nanoparticles and small‐molecule dyes, have been employed to label and monitor injected stem cells.^185^ This preloading of cells prevents macrophage phagocytosis of the nanoparticles.^186^ Organic semiconducting nanoparticles in the far infrared window enhance photoacoustic contrast, allowing for real time monitoring of stem cell behaviors like differentiation. This could advance the understanding of stem cell‐based therapy.^187^ Recent developments show that PAI is effective in assessing the immune response during treatment of cancer. In particular, using metal–organic frameworks (MOFs) as photoacoustic contrast agents has shown potential in tracking tumor‐associated macrophage (TAM) re‐education during treatment. Z. Fan et al. designed MOF‐based nanoparticles loaded with Caspase‐1 for releasing their cargo in TME, and R848 for targeting M‐like TAM to induce re‐education. In this work, a caspase‐1 nanoreporter (MCNR) based on MOFs was developed to deliver a TLR7/8 agonist specifically to TAMs. This nanoreporter enabled the detection of caspase‐1 activity, which is an indication of immunological reprogramming, using both photoacoustic and FLI. The MCNRs show considerable tumor targeting and increased accumulation in the tumor location, allowing for accurate imaging and effective treatment.

**FIGURE 9:**
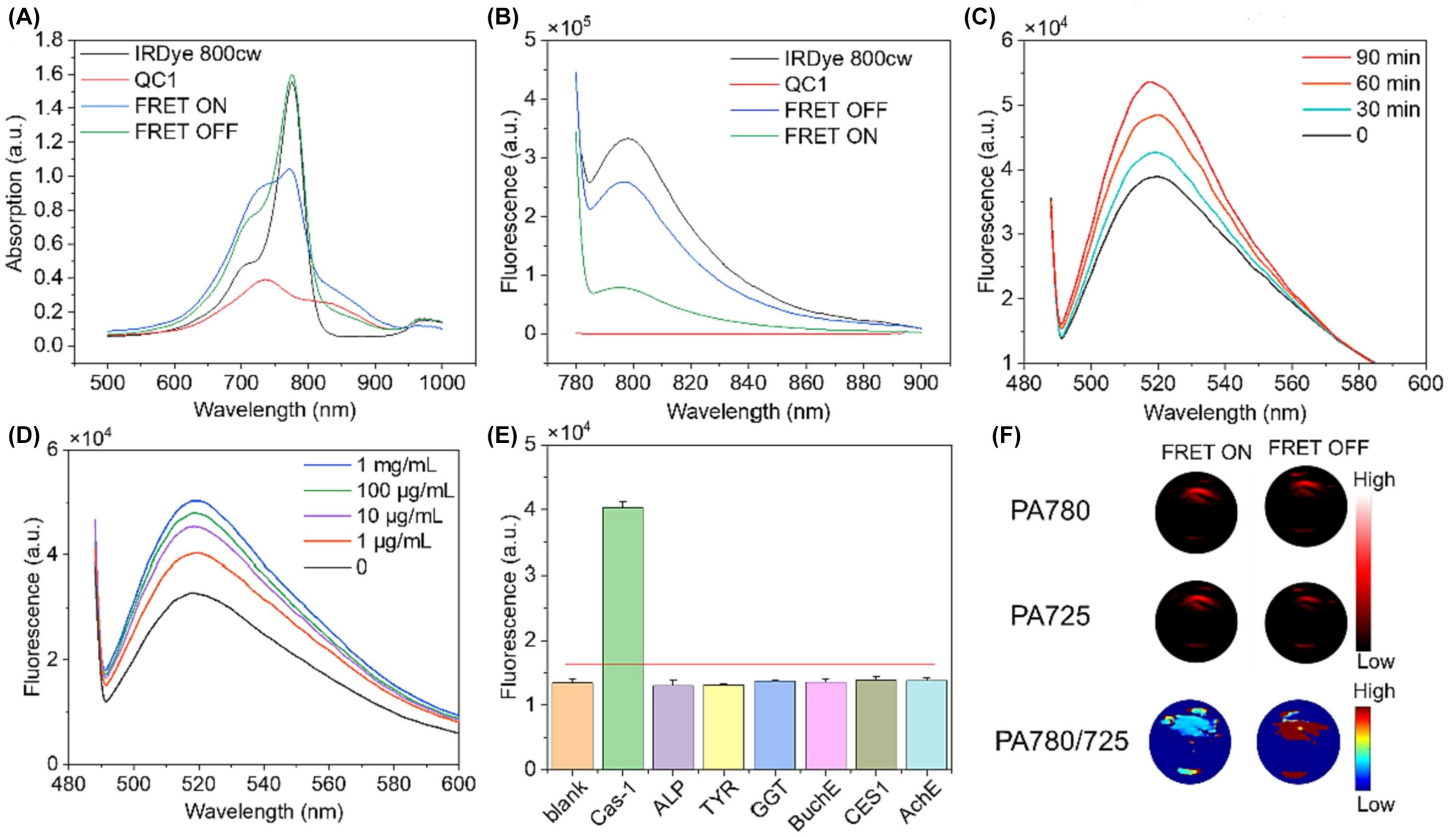
(A) and fluorescence spectrum (B) changes of Caspase‐1 imaging probe before and after activation. (C) Time‐dependent probe responsiveness. (D) Caspase‐1 concentration‐dependent probe responsiveness. (E) Specificity of Caspase‐1 imaging probe. (F) In vitro PA effect of Caspase‐1 imaging probe.^188^

**FIGURE 10:**
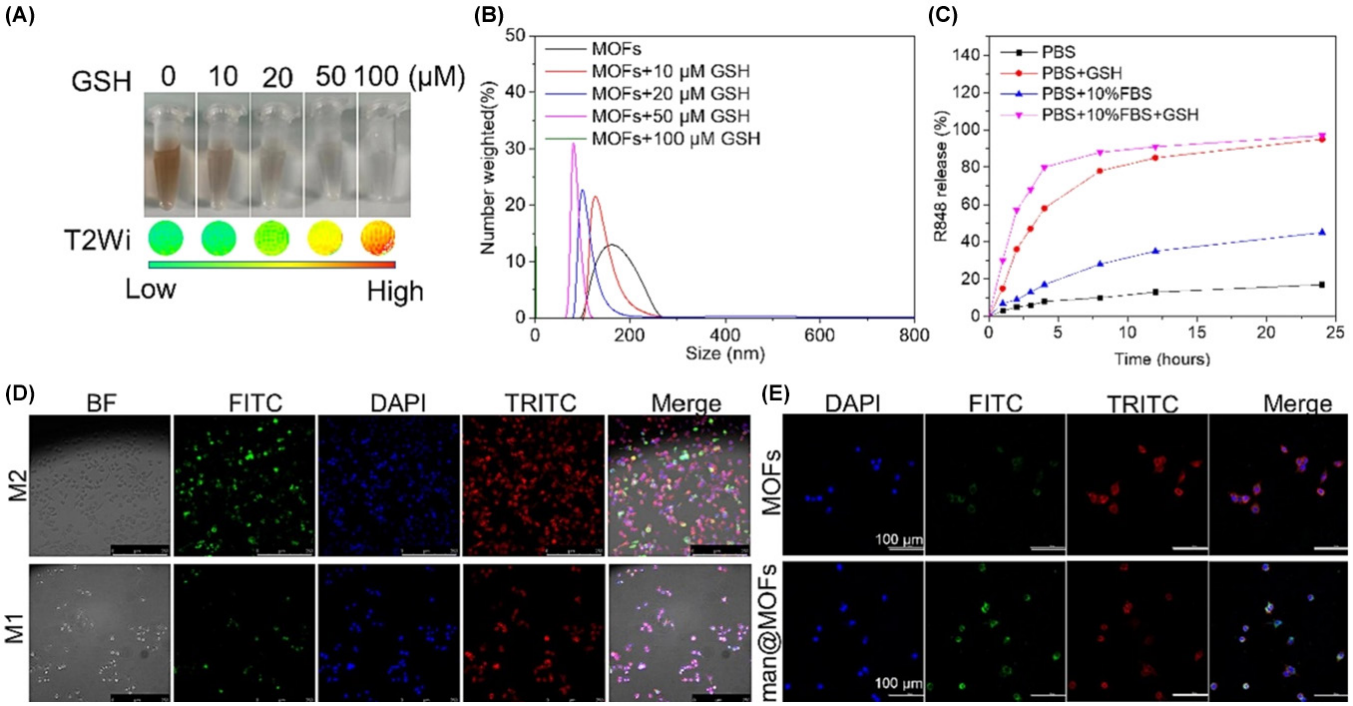
(A) Magnetic resonance imaging was used to detect GSH‐triggered degradation of nanoparticles. (B) Particle size change in response to GSH. (C) Accumulation curve of drug release. (D) Fluorescence imaging was used to observe the targeting effect of MCNRs on M1 and M2 macrophages. Scale bar: 250 μm. (E) Fluorescence imaging was used to observe the targeting effect of MIL101‐Fe and mannose labeled MIL101‐Fe to M2 macrophages.^188^ Scale bar: 100 μm.

The experimental results showed that PAI is capable of effectively tracking TAMs polarization state in vivo. Researchers developed a subcutaneous tumor model to use it for the photoacoustic/fluorescence dual‐modal imaging to investigate TAM polarization. The study found that MCNRs dramatically boosted caspase‐1 activity in TAMs within the tumor location, as shown by strong fluorescence and photoacoustic signals. This dual‐modal method show complete information on the geographical and temporal dynamics of TAM re‐education (Figure 9). Thus, in vivo experiments on tumor‐bearing mice show that treatment with MCNRs resulted in notable tumor suppression and higher survival rates than control groups. Immunohistochemically examination demonstrated that MCNR therapy boosted CD8+ T cell infiltration in the tumor microenvironment, indicating a strong antitumor immune response (Figure 10).

PAI is an effective method for real time monitoring of immunotherapy in the TME. Its ability to offer high‐resolution, functional images deep inside tissues makes it useful for assessing the efficacy of immunotherapeutic drugs and understanding their mechanisms of action. The integration of PAI with advanced nanomaterials such as MCNRs defines an important advancement in cancer theranostic, offering better outcomes for patients.^188^

### 6.5 Nanomaterial‐based immunotherapy optical imaging

Current advances in immunotherapy have opened up possibilities for creating therapies with diminished side effects. Despite these advances, the TME exerts significant suppressive effects that lead to suboptimal clinical outcomes with existing cancer immunotherapies. Precise tumor imaging is crucial for designing effective treatments, including immunotherapy, as shown in Figure 12. There have been developments in utilizing nanomaterial‐based approaches to manipulate the TME, aiming to enhance robust immunotherapeutic responses.^189^ Nanoparticles are of paramount importance in the realm of immunotherapy.^39^ Advancements in nanotechnology have emerged as a promising prospect, serving as a beacon of hope that fuels research interest in utilizing nanoparticles in the making of innovative instruments for cancer therapies and cancer imaging.^190^, ^191^, ^192^, ^193^, ^194^, ^195^, ^196^, ^197^, ^198^, ^199^ The beneficial aspects and inevitable pitfalls of the recent preclinical and clinical use of immunomodulatory therapies using nanoparticles (NPs) alone or in combination are shown in Table 2. In biological and biomedical fields, optical imaging nanoprobes and fluorescent imaging nanoprobes have been applied, and after the reaction with different targets or stimuli, the fluorescent signals can be observed by the detection system at a high resolution.^200^ In this section, we review recent advancements in immunotherapy imaging. Nanoparticles are employed to target, detect, stimulate, and administer therapeutic doses in vivo. This approach enables precise, depth‐resolved tracking of immunomarkers throughout the entire body, ensuring high accuracy in pre and posttreatment assessments.^39^

As the field of immunotherapy and biotechnology advances, there is an increasing need for efficient optical imaging methods. These techniques are important for understanding how the immune system, diseases, and immunotherapeutic agents interact. They help to improve the outcomes of immunotherapy.^62^ To assess the progression of tumor‐associated macrophage (TAM) infiltration in the course of tumorigenesis and their reaction when under attack, recent advancements in in vivo imaging technologies have been employed. For instance, the macrophage phagocytic response in the context of the anti‐tumor effects following the disruption of SIRPα‐CD47 has been investigated utilizing in vitro live microscopy and in vivo bioluminescence access diverse tumor types.^165^, ^201^, ^202^, ^203^, ^204^ Moreover, fluorescently labeled macrophages that have undergone genetic modification were employed to monitor the anti‐CD47 blockade in brain tumors.^205^ The Weissleder group recently published a study on the in vivo imaging of TAMs throughout immunotherapy, utilizing cell‐specific techniques.^160^, ^204^

**FIGURE 11:**
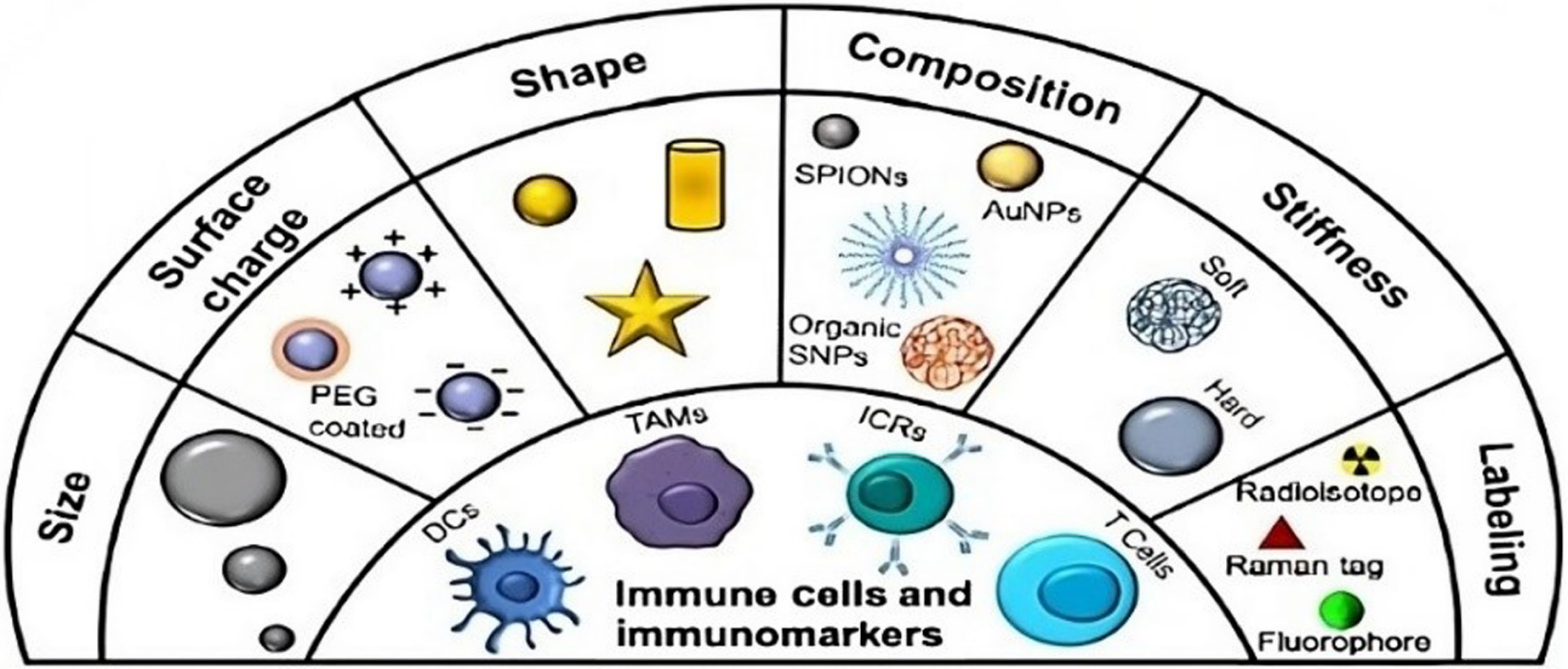
Here are the key factors to be considered when developing nanoparticles: These factors include material composition, surface charge, size, shape, and stiffness. The purpose of integration is to aid immune cells and immunomarker imaging, thereby enabling image‐guided immunotherapies. The abbreviations used in this context are as follows: DCs for dendritic cells, Au NPs for gold nanoparticles, PEG for polyethylene glycol, ICRs for immune checkpoint receptors, SPOINs for super‐paramagnetic iron oxide nanoparticles, and TAM for TAMs. Reproduced with permission from ref.^39^ Copyright 2019, Elsevier.

Nanotechnology can help with potential cancer therapies, the modulation of TME, and the selection of imaging modalities for cancer diagnosis and treatment monitoring.^62^ Table 3 presents an extensive compilation of intelligent nanoparticle‐based detection methods for diverse immune cells with the consistent imaging tools employed. Besides, fundamental design considerations for these nanoparticles, such as surface properties, composition, shape, and size, are assessed for their applicability within TME, as depicted in Figure 11.^39^

**TABLE 3:** Preclinical or clinical nanoparticle agents are currently used to modulate the tumor immune microenvironment.^109^


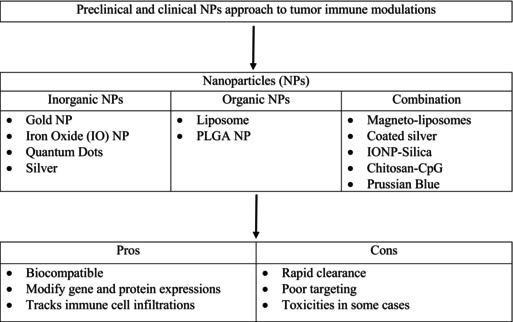

**FIGURE 12:**
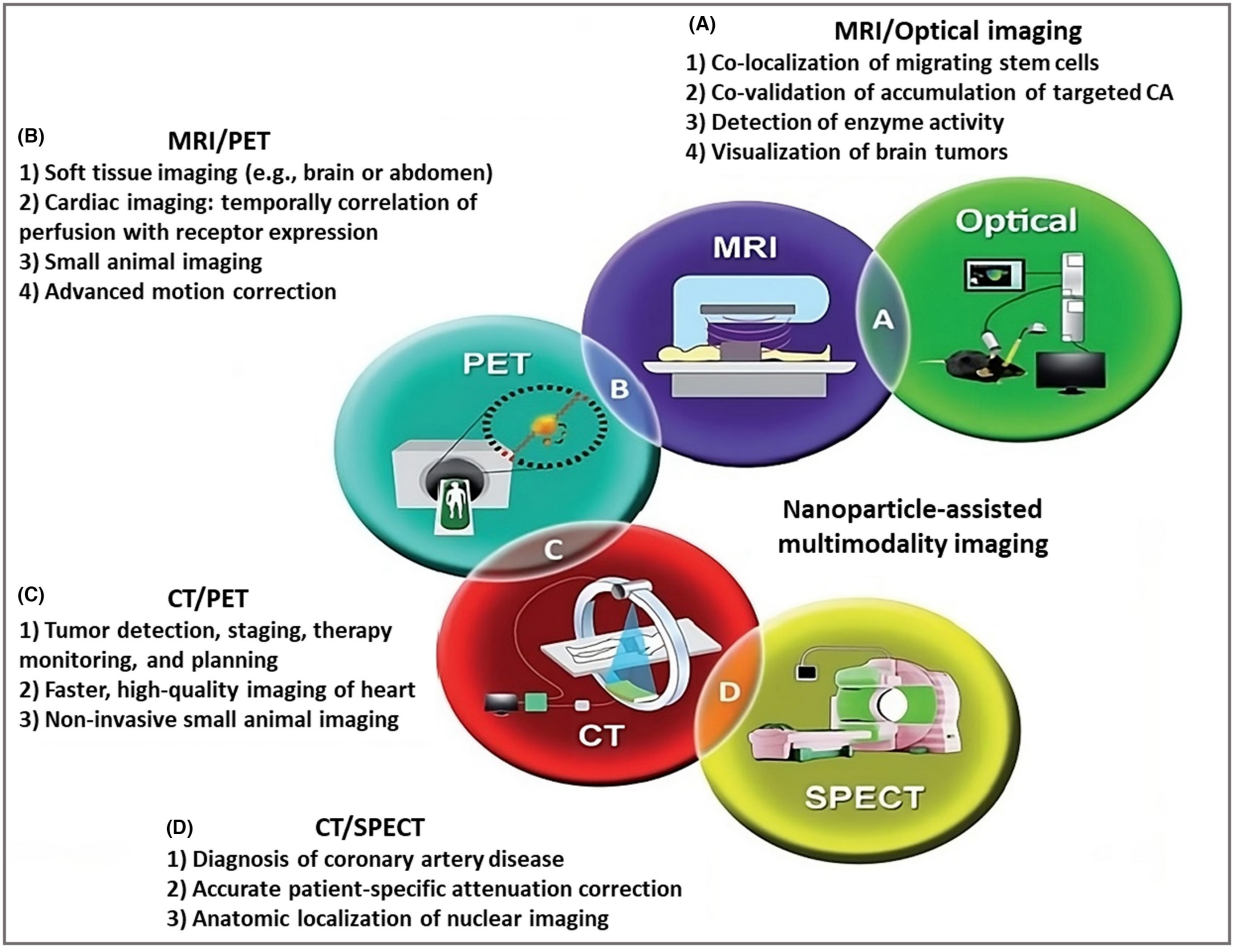
A schematic illustration of multimodality imaging utilizing nanoparticles. In the realm of in vivo noninvasive imaging technologies, hybrid tracers play a crucial role in diverse imaging schemes and uses, comprising (A) MRI/Optical Imaging, (B) MRI/PET, (C) CT/PET, (d) CT/SPECT. The abbreviations denote PET, Positron emission tomography; CT, Computed tomography; SPECT, Single‐photon emission computed tomography. Reproduced with permission from ref.^62^ Copyright 2017, Future Medicine.

**FIGURE 13:**
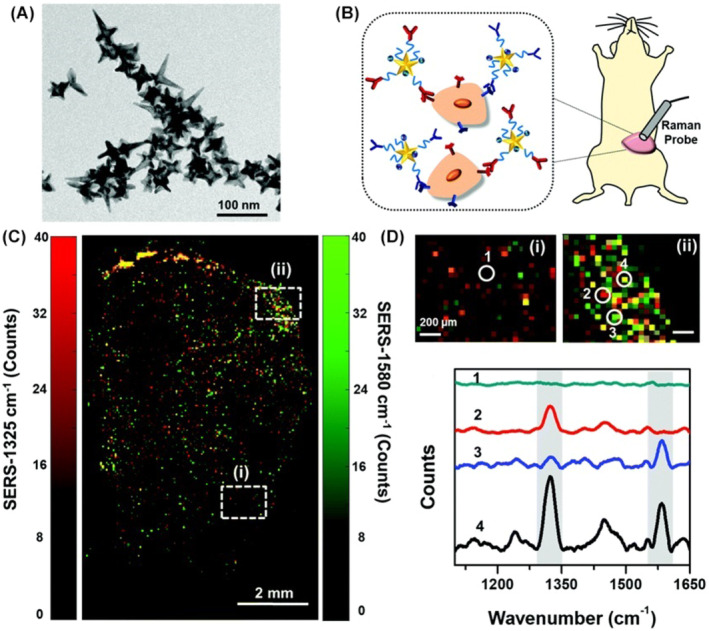
Utilizing gold nanoparticles for immune‐imaging purposes. (A) The Au nano‐stars (Au NSs) have an anisotropic morphology observed via transmission electron micrograph. (B) Mice‐bearing mammary tumors were administered a combination of anti‐epidermal growth factor (EGFR)‐p‐mercaptobenzoic acid (pMBA)‐Au NS and anti‐PD‐L1‐5,5′‐dithiol‐bis (2‐nitrobenzoic acid) (DTNB)‐Au NS for the concurrent detection of both markers. (**C**) Multiplexed detection is illustrated in an ex‐vivo SERS map of entire tumor lesions. (D) Zoomed‐in ex vivo Raman maps and corresponding Raman spectra highlight regions with (1) no SERS signal, (2) PD‐L1‐rich tissue, (3) EGFR‐rich tissue, and (4) both receptors. Adapted with permission from.^223^ Copyright 2018, Royal Society of Chemistry.

**FIGURE 14:**
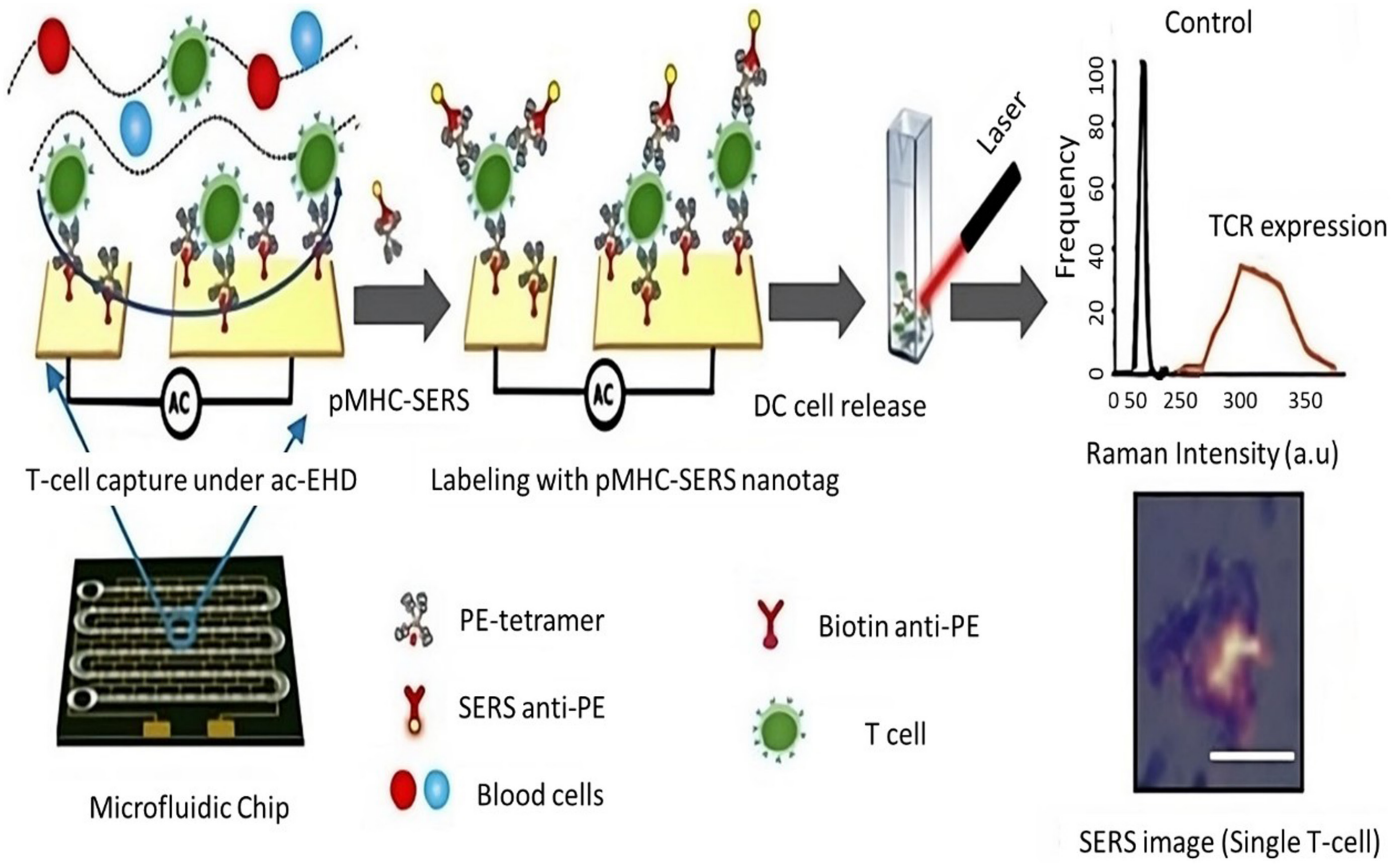
A SERS‐microfluidic platform has been developed to integrate antigen‐specific T‐cell isolation and SERS mapping of T‐cell receptor expressions. Reproduced with permission from ref.^242^ Copyright 2018, Elsevier.

Nanoparticles, which are less than 100 nm in size, can consist of materials with a biochemical nature, including metal oxides, metal silicates, liposomes, polymers, biomolecules, carbon, and dendrimers.^206^, ^207^, ^208^ Nanoparticles have many factors to consider, such as surface chemistry, size, shape, targeting specific biomolecules, methods of delivery, protein corona formation,^209^ and intercellular targeting.^210^ Maeda et al. validate the augmented permeability and retention effect by facilitating nanoparticle extravasation via fenestrated blood vessels. This process led to the accumulation of the nanoparticles, enabling targeted drug delivery for precision on‐site localization and unveiling novel therapeutic prospects.^211^, ^212^ Several conceivable interactions occur between nanoparticles and the nearby environment in various cell types. These interactions give rise to different outcomes, including the modification of signaling pathways and the disturbance of cells by infecting viruses, as well as influence on proteins, secreted molecules, and chromatin complexes within cells.^213^, ^214^ The immune system can be influenced by the physical characteristics of nanoparticles, leading to effects such as immunosuppression, hypersensitivity, immunogenicity, and autoimmunity.^215^ Hence, nanoparticles can elicit innate and adaptive immune reactions, either through direct means or indirectly, such as necrotic dying cells.^216^

It is important for accurate evaluation of how tumors and the TME respond to treatments like immunotherapy for successful treatment development and execution. Optical imaging offers the advantage of monitoring the dynamics of immune cells, providing valuable insights into immunotherapeutic responses. For example, the spatiotemporal dynamics of immunocytes were studied in the context of combined immunotherapy involving adoptive cell transfer (ACT) and cyclophosphamide for melanoma treatment.^217^ An alternate method employed the assessment of the antibody‐focused nano‐drug Au‐SM5‐1 for hepatocellular carcinoma (HCC) treatment through bioluminescence tomography. This nano‐drug slowed cell proliferation and caused apoptosis in both in vitro and in vivo.^218^ Furthermore, the efficacy in treating gastric cancer has been demonstrated through real time in vivo imaging labeling of allogenic DCs with NIR fluorescent quantum dots. This approach, when combined with tumor cell‐fused vaccines and cytokine‐induced cell killing, proved to be effective.^219^ Different approaches were employed to find out the most favorable timing for in vivo ACT targeting glioma xenografts that express EGFRvIII. This involved monoclonal antibody use against EGFRvIII (biotin‐4G1) for the specific targeting of chimeric antigen receptor CAR‐T therapy.^220^ Furthermore, the effective stimulation of antigen cross‐presentation to OVA‐specific CD8^+^ T cells in lymph nodes was verified by NIR fluorescent dyes associated with nanoparticles composed of poly (lactide‐cohydroxymethyl glycolic acid) OVA.^221^ In a study utilizing a human colon cancer model (HTC‐116), the efficacy of the cetuximab therapy response was demonstrated through the NIR fluorescence of vascular endothelial growth factor (VEGF) expression. Employing ranibizumab with a dye conjugate of an anti‐VEGF antibody Fab fragment, namely Dye755‐Ran, achieved this. The findings underscore the effectiveness of NIR fluorescence for assessing response to cetuximab treatment in this specific cancer model.^222^ A precise assessment of the response to immunotherapy holds significant importance, especially during the clinical phase.^62^

Gold nanoparticles are widely used for immune imaging because they are safe, compatible with the body, and easy to make. These qualities allow for the alteration of design parameters and the association of NIR light absorption properties, as required.^39^ Bardhan and coworkers illustrated the importance of employing active targeting agents. Utilizing gold nanoparticles conjugated with receptor‐specific antibodies, they achieved multiplexed detection (as depicted in Figure 13A,B). This approach enables simultaneous identification of PD‐L1 and epidermal growth factor receptor (EGFR) using SERS in both in vivo^223^ and in vitro^224^ settings for breast cancers. Ex‐vivo SERS maps at the cellular level reveal a heterogenous distribution of PD‐L1 and EGFR throughout entire lesions, as depicted in Figure 13C,D. This assessment, conducted on breast tumors, demonstrates the presence of regions rich in PD‐L1 and EGFR within tumors, as identified through higher magnification SERS.^39^

SERS is an essential optical sensing technology used for the noninvasive detection of biomolecules, including proteins. SERS measures the inelastic light scattering of molecules with higher increasing signals ($\geq 10^{8}$) when the molecules are close to Ag, Au, plasmonic metallic nanoparticles, and nanostructures.^225^ These results are promising, as the integration of nanoparticles with controlled shapes and potent imaging, modality aids a robust correlation between in vivo signals and ex vivo study. This correlation is crucial for translating particular expertise into patient stratification for immunotherapies. Technology like optical imaging has provided detailed information about the components of the TME, leading to the development of new nano‐formulations to advance immunotherapy. Advancements in technology, such as optical imaging, have offered a thorough perspective on the components of the TME,^226^ fueling the creation of innovative nano‐formulations to progress immunotherapy. Recent strides in technology, such as optical imaging, have yielded a thorough understanding of the TME, thereby catalyzing the creation of innovative nano‐formulations to enhance the field of immunotherapy.^189^

### 6.6 SERS nanomaterial‐based study of immunotherapies

The results of immunotherapy can vary among patients due to the presence of different immune evasion pathways. Consequently, it is necessary to profile biomarkers in the TME to predict the immunotherapy efficacy in responders and nonresponders.^227^, ^228^, ^229^, ^230^, ^231^, ^232^ The ongoing advancements in SERS‐active nanomaterials, aimed at enhancing the sensitivity and multiplicity of SERS immunomarker detection, have garnered considerable interest. SERS employed for labeling targeted biomolecules through specific interactions during detection, encompassing laser activation and signal acquisition on metallic nanomaterials modified at the surface.^233^, ^234^, ^235^ Nanomaterials that are active in SERS show remarkable efficiency in enhancing Raman signals. These materials are equipped with diverse Raman reporter molecules, allowing multiplexed detection of a range of biomolecules through inventive quantification techniques.^225^, ^236^, ^237^, ^238^, ^239^ Thus, the combination of spectroscopic effects and cutting‐edge nanomaterials has enabled SERS within compact reaction volumes to exhibit outstanding detection capabilities.^240^ Stepula et al. presented a technique for examining PD‐L1 biomarker expression on the surfaces of cancer cells using a SERS approach based on Au/Au core/satellite nanoparticles. The strength of Raman signatures of the reporter molecules linked to antibody‐functionalized Au/Au core/satellite structure. Enhancement of the Raman signal was affected via the plasmonic coupling between Au core and Au satellites, leading to the creation of highly localized electric fields, referred to as “spots,” which contributed to the amplification of the Raman signal.^241^

In the adaptive immune response in the TME, understanding various types of T cells in tumor areas plays a dynamic part in describing new drug target choice, host immune response, and therapy valuation. Dey et al. created a sensitive method that controls microfluidics for target antigen‐specific T cell isolation and SERS‐active nanomaterials for uncovering T cell receptor expression heterogeneity at the individual cell level, as shown in Figure 14.^242^ Immunoimaging of multiple TME components is crucial for understanding how immunotherapy works. It helps us to predict how well the treatment will work and keeps track of how effective it is.^243^, ^244^ The benefits comprise unusual spatiotemporal resolution, multiplexing with negligible spectral overlapping, and specific Raman reporter labeling of targets.^245^, ^246^, ^247^ Bardhan and coworkers also confirmed progressive studies for SERS imaging of checkpoint inhibition immunomarkers on utilizing gold nanostructures.^248^

## 7 FUTURE OUTLOOK

Researchers still need to overcome many challenges, like the variability in sensitivity and specificity of optical imaging techniques and limited depth of penetration into tissues. This makes it difficult to visualize the TME in large and deep tumors. In addition, various factors, such as auto‐fluorescence and tissue scattering, can affect optical imaging, making it challenging to interpret images. Research in the field of optical imaging for TME immunotherapy evaluation is ongoing, and promising areas of research include:

- Developing new optical imaging techniques that can image deeper into tissue with higher resolution.
- Development of new optical imaging probes that are more sensitive and specific to different aspects of the TME.
- Further research is required to classify and develop present biomarkers and general imaging techniques for better clinical monitoring. Although further optimization is essential, utilizing imaging to understand and improve immunotherapy for TME is promising.^62^
- However, the nanoparticle stiffness to cellular uptake remains poorly understood within the immune microenvironment. Organized training is required to determine how they can optimize to design of the nanoparticle library for immunotherapy.^39^
- Using the (NIR‐II) optical imaging technology with deeper tissue penetration and less auto‐fluorescence will endorse theranostic efficacy, better understand the clearer visualization of small tumor lesions and metastatic lesions and improve efficient imaging‐guided tumor treatment.
- Future investigations may emphasize numerous nano‐systems with more complicated architectures that combine diagnosis and treatment and understand the mechanism of functional nanomaterials for treating cancer.^177^

## 8 CONCLUSIONS

Optical imaging is a powerful tool for evaluating immunotherapy in the TME with enhanced precision owing to its advanced monitoring speed, resolution, and sensitivity. It has been used to image various characteristics of the TME, including immune cell infiltration, vasculature, and hypoxia, before, during, and after immunotherapy. Researchers are developing optical imaging probes with high specificity and sensitivity for targeting specific cell types, proteins, and other molecules of interest. This allowed the visualization and quantification of cellular and molecular processes in the TME in unprecedented detail. Optical imaging plays a crucial role in clinical trials by tracking patient responses to immunotherapy, and it will likely play a significant part in improving the selection of patients for immunotherapy, monitoring the response to treatment, and identifying new therapeutic targets.

## AUTHOR CONTRIBUTIONS

**UM E KALSOOM:** Writing – original draft (lead). **Shiqi Wang:** Visualization (supporting). **Junle Qu:** Visualization (supporting). **Liwei Liu:** Supervision (supporting).

## FUNDING INFORMATION

Development Program of China (2021YFF0502900), the National Natural Science Foundation of China (62225505/61935012/62175163/61835009/62127819/62205220/892004043), The National Key Research and Shenzhen Key Projects (JCYJ20200109105404067), Shenzhen Talent Innovation Project (RCJC20210706091949022), Shenzhen Science and Technology Planning Project (ZDSYS20210623092006020).

## Data Availability

N/A.
